# ﻿Mapping the distribution of armored harvestmen (Opiliones, Laniatores) in Colombia: updated list of species, taxonomic contributions, and insight of diversity in protected areas

**DOI:** 10.3897/zookeys.1175.102485

**Published:** 2023-08-17

**Authors:** Osvaldo Villarreal, Daniela Ahumada-C., Leonardo Delgado-Santa

**Affiliations:** 1 Departamento de Invertebrados, Museu Nacional/Universidade Federal do Rio de Janeiro, Quinta da Boa Vista, São Cristóvão, 20.940-040, Rio de Janeiro, RJ, Brazil; 2 Museo del Instituto de Zoología Agrícola, Facultad de Agronomía, Universidad Central de Venezuela, Maracay, Aragua, Venezuela; 3 Grupo de Investigación Biología Descriptiva y Aplicada, Programa de Biología, Universidad de Cartagena, Cartagena de Indias, Bolívar, Colombia; 4 Grupo de Investigación Hidrobiología, Programa de Biología, Universidad de Cartagena, Cartagena de Indias, Bolívar, Colombia; 5 Grupo de Investigación Ecdysis, Programa de Biología, Universidad del Quindío, Armenia, Quindío, Colombia

**Keywords:** Checklist, ecoregions, harvestmen, Neotropics, protected natural areas

## Abstract

Colombia is a biodiverse country with 1,486 protected areas covering almost 50 million hectares, yet little is known about the biodiversity they harbor, particularly in terms of harvestmen (Arachnida: Opiliones). This study provides a comprehensive updated summary of the armored harvestmen (Laniatores) species found in Colombia with a list of 173 nominal species, focusing on the Laniatores fauna found in protected areas and its diversity is detailed and analyzed. Maps with all records associated with ecoregions and protected areas in Colombia are presented. Additionally, three new Laniatores species are described here: *Phalangodusandresi***sp. nov.** from the department of Cundinamarca, and *Ventrifurcaphallaina***sp. nov.** and *Ampycellafortunata***sp. nov.** from the department of Valle del Cauca; and a new family assignment is proposed for *Paraphalangodus* Roewer, 1915, placing it in the family Nomoclastidae. Information available on Laniatores in the National System of Protected Areas is still scarce and promoting strategies to facilitate the regulatory procedures for collecting specimens in these areas and increasing investment in basic science projects, are suggested to improve the understanding and study of the Laniatores fauna and other invertebrates in Colombia. Finally, a chronicle and timeline set of figures of species of Laniatores from Colombia, described by various authors during three periods, is given.

## ﻿Introduction

Colombia is one of the most biodiverse countries in the world (Andrade-C 2011). This diversity is represented and legally protected in 1,486 conservation units (covering 49,884,326.77 hectares), under the figure of National System of Protected Areas (RUNAP 2022). These areas allow ecological self-regulation, their ecosystems have not been substantially altered by human exploitation or occupation, and their biodiversity is under management (CONPES 2021). Although Colombia is one of the countries in South America with the largest number of areas under conservation (CONPES 2021), knowledge about the species it harbors is scarce, especially in terms of harvestmen (Arachnida: Opiliones). Colombia ranks third in South America in terms of the number of recorded Opiliones species, only behind Brazil (1,010) and Venezuela (392) (Villarreal et al. 2021a). The first effort to list the arachnid species of Colombia was made by Flórez and Sánchez-C (1995), summarizing 77 species of harvestmen. Later, all records and distribution data on Colombian Laniatores, which constitute the vast majority of Opiliones in the country, were compiled by Kury (2003) into an excellent New World Laniatores catalog. Taxonomic studies on harvestmen have become increasingly frequent in the Neotropics, especially in Colombia, where numerous genera and species have been recently described or recorded (e.g., Villarreal and Rodríguez 2006; García 2014; Villarreal and García 2016; Pinzón and Pinto-da-Rocha 2020; Villarreal and García 2021). More recently, a general diagnosis of the current knowledge of the group in Colombia was published (Perafán et al. 2013), recording a total of 162 Opiliones species in the country. Minor local inventory works mention 170 (García and Medrano 2015) and 186 (Barriga et al. 2019) species of Opiliones in Colombia. Only two opilionofaunal inventory and/or ecological studies have been conducted in Colombia to our knowledge so far (García and Medrano 2015; De Moya et al. 2021). García and Medrano (2015) carried out the guide of Opiliones of the Reserva Natural Rio Ñambí, a protected area on the coastal foothills (department of Nariño) (CONPES 2021), recording 12 families and a wealth of undescribed species. More recently, De Moya et al. (2021) studied the diversity of Opiliones by altitude in Sierra Nevada de Santa Marta (department of Magdalena), an area of high conservation importance due to its significant climatic variability and high rates of endemism, recording nine families. Studies focused on analyzing the composition of neotropical harvestmen in protected areas are not frequent. These have been carried out mainly in Brazil, among them, Soares and Soares (1970) describe seven species for the Itatiaia Biological Station (Rio de Janeiro); Adis et al. (2002) record seven species for the Ducke Reserve (Amazonia), and Bragagnolo and Pinto-da-Rocha (2003) recorded 52 species in the Serra dos Órgãos National Park (Rio de Janeiro). A recent precedent was made by Guerrero (2019), who recorded 17 species from 30 protected areas in the province of Buenos Aires (Argentina). However, like the situation in Colombia, he concludes that many important areas still remain for the conservation of diversity that have yet to be surveyed to understand their harvestmen composition.

Since the publication of the New World Laniatores catalog by Kury (2003), more than 20 years ago, Colombia has undergone numerous changes and additions to its opilionofauna. This is partly due to the appearance of a growing group of enthusiastic local and regional arachnology researchers who have turned their interest to the fauna of this country, as well as the expansion of its biological collections and collaboration with other renowned institutions. Currently, Colombia has 162 species of armored harvestmen (Opiliones: Laniatores) recorded, belonging to 12 families of which 46 species have been recorded or described after 2003. Therefore, we consider it opportune to provide an updated and detailed summary of the taxonomic changes in the Colombian laniatorean fauna after 2003.

The purpose of this paper was to provide an updated list of species and summarize the taxonomic changes and additions that occurred after 2003. Additionally, we aim to relate the distribution of Colombian species with the biogeographical areas proposed by WWF, discuss the presence of species in the country’s National System of Protected Areas (NSPA), and present the descriptions of two new cranaid species from the departments of Cundinamarca and Valle del Cauca as well as one new gonyleptid species from the department of Valle del Cauca.

### ﻿Taxonomic background for *Phalangodus*, *Ventrifurca*, and *Ampycella* of Colombia

The neotropical family Cranaidae has attracted attention from taxonomists in recent years; nevertheless, the definition of generic or suprageneric groups in this family remains unsatisfactory (Kury and Villarreal 2015; Villarreal and García 2016). Although some groups have been reviewed, leading to the resolution of generic synonyms, new combinations or redefinitions, improper placements, as well as descriptions of new species (e.g., Orrico and Kury 2009; Pinto-da-Rocha and Bonaldo 2011; Hara et al. 2014; Villarreal et al. 2015; Villarreal and García 2016; Hara et al. 2017), much work remains to be done.

Only three works have analyzed the phylogenetic relationships within the family Cranaidae and the genus *Phalangodus* Gervais, 1842 has only been studied in a phylogenetic context in an analysis of the familial relationships of Gonyleptoidea (Kury and Villarreal 2015), where it appears as sister group to the other four representatives of the family. Despite this, the taxonomy of *Phalangodus* is relatively well understood, with a total of six species described, five of them known from Colombia (Villarreal and García 2016). A similar case exists for the genus *Ventrifurca* Roewer, 1913, which was recently reviewed (Villarreal et al. 2015), revealing some synonyms and the discovery of a new species from the department of Quindío. Currently, three species of *Ventrifurca* are known from Colombia, distributed in the western Andes.

Two species have been recorded from Colombia, belonging to the family Ampycidae, *Ampycustelifer* (Butler, 1873), and *Licornustama* Villarreal & Kury, 2012 (García 2014). This family lacks taxonomic revisionary work, and its genera are poorly delimited or defined. Recent works have contributed to better understanding the diversity of some genera within Ampycidae (e.g., Tourinho and Mendes 2014; Hara et al. 2017) or described new species (Villarreal and Kury 2012). The genus *Ampycella* Roewer, 1929 includes two Andean species from Ecuador (Kury et al. 2022), vaguely described and illustrated and whose genital morphology is unknown.

This paper describes three species: two new cranaid (*Phalangodus* and *Ventrifurca*) and one ampycid (*Ampycella*, including the first image of the male genitalia for the genus), collected in the departments of Cundinamarca in the Central Mountain Range in the Colombian Andes and the department of Valle del Cauca in Chocó Biogeographic region.

## ﻿Materials and methods

### ﻿Taxonomy

Individuals of each species were photographed using a Leica M205C stereoscope attached to a Leica DFC450 digital camera, a Wild Heerbrugg stereoscope attached to a Nikon COOLPIX P900 and a Wild Heerbrugg microscope attached to a HAYEAR 2307 digital camera. The multiple resultant images at different focal planes were combined with Combine ZP Suite software (Hadley 2015) to increase the depth of field and were thereafter edited in Photoshop CC 2014 software. Drawings of the species were made using Illustrator CC 2017 and Inkscape 1.2.2 softwares (Harrington 2004–2005). To color descriptions the standard names of the 267 Color Centroids of the NBS/IBCC Color System were used as named in Centore (2016). Male genitalia were studied using standard methods for this structure (Acosta et al. 2007).

Morphological terminology and patterns of taxonomic description follow Villarreal and García (2016) with slight modifications; integumentary ornamentation follows DaSilva and Gnaspini (2010); terminology for chaetotaxy of penis ventral plate follows Kury and Villarreal (2015) with the modifications proposed by Villarreal and García (2016) for the genus *Phalangodus*; the ovipositor morphology follows Villarreal and García (2016); terminology of dorsal scutum outline types follows Kury and Medrano (2016).

The first-order administrative divisions of Colombia (departments) are underlined. Maps were made using ArcGIS^®^ 10.1 software (ESRI 2022). Colored areas represent WWF Terrestrial Eco-regions of the World (Olson et al. 2001), here abbreviated as WWF.

Morphometric abbreviations are: **AL** = maximum abdominal scutum length; **AW** = maximum abdominal scutum width; **BaCh** = basichelicerite length; Cl = claw; **CL** = carapace length; ClPp = pedipalp claw; **CW** = maximum carapace width; **DS** = dorsal scutum; **DSL** = dorsal scutum length; **Fe** = femur; FeL I = femur length I; FeL II = femur length II; FeL III = femur length III; FeL IV = femur length IV; **IOD** = inter ocular distance; **MS** = macrosetae of penis; **Mt** = Metatarsus; ; **Pp** = pedipalps; **FePp** = pedipalpal femur; **PaPp** = pedipalpal patella; **TaPp** = pedipalpal tarsus; **TiPp** = pedipalpal tibia; **Ta** = tarsus; **Ti** = tibia; TiL I = tibia length I; TiL II = tibia length II; TiL III = tibia length III; TiL IV = tibia length IV; **VP** = ventral plate. All measurements are in mm unless otherwise noted. The material studied is deposited in the arachnological collections of Instituto de Ciencias Naturales (**ICN**),Universidad Nacional, Bogotá, Colombia; Colección de Insectos de la Universidad del Quindío (**CIUQ**), Armenia, Colombia; Museo del Instituto de Zoología Agrícola “Francisco Fernández Yépez” (**MIZA**), Maracay, Venezuela; and Museu Nacional, Universidade Federal do Rio de Janeiro (**MNRJ**), Rio de Janeiro, Brazil.

### ﻿Species inventory

A comprehensive list of all described Laniatores species occurring in Colombia was compiled, based on the bibliography published until April 2023, including all valid species of Laniatores recorded for Colombia. The family and superfamily classification follows Kury et al. (2022). The logonymic information is updated only for Colombian species that were described, recorded, or underwent nomenclatural changes after New World Laniatores catalog (Kury 2003). The taxonomic references in the logonymy of the listed species were not considered in the References section.

A list of all protected areas under the NSPA jurisdiction, which harbor records of Laniatores species, was compiled. Only areas with some form of public regulation for their protection were considered. For each protected area, the families and species of armored harvestmen recorded were listed (Table 1). The source of the species data can be seen in the Suppl. material 1.

**Table 1.** Opiliones species by family recorded in RUNAP from Colombia.

Category	RUNAP	Family	Species
Regional Integrated Management Districts (RDIM)	Cuenca alta del Río Atrato	Manaosbiidae	*Camelianusfuhrmanni*
	de la cuenca alta del Río Quindío de Salento	Cranaidae	*Allocranauscolumbianus*
		Nomoclastidae	*Quindinabella*
		Stygnidae	*Eutimesiusephippiatus*
	Delta del Río Ranchería	Cranaidae	*Cranausalbipustulatus*
	Paramo de Guargua y laguna Verde	Cosmetidae	*Rhaucusserripes*
	Paramo de guerrero	Cosmetidae	*Rhaucusquinquelineatus*
	Páramos de Guantiva y la Rusia	Cosmetidae	*Rhaucuspapilionaceus*
	Río Rubachoque y pantano de arce	Cosmetidae	*Rhaucusvulneratus*
	San Miguel	Cranaidae	*Megacranauspygoplus*
	Sector Salto del Tequendama y Cerro Manjui	Agoristenidae	*Muscopilioonod*
		Cosmetidae	*Eulibitiamaculata*
			*Rhaucusserripes*
	Serranía de los Yariguies	Agoristenidae	*Leptostygnusyarigui*
		Cosmetidae	*Rhaucuspapilionaceus*
		Cranaidae	*Phalangodusbriareos*
	Serrania de Perija	Agoristenidae	*Avimatroglobia*
			*Avimavenezuelica*
National Natural Parks (NNP)	Chingaza	Cosmetidae	*Libitiabipunctata*
	Paramillo	Agoristenidae	*Avimatuttifrutti*
	Sierra de la macarena	Cosmetidae	*Meterginusprosopis*
		Cranaidae	*Phareicranausangelicus*
	Sumapaz	Cosmetidae	*Eulibitiamaculata*
			*Rhaucusvulneratus*
		Fissiphalliidae	*Fissiphalliusspinulatus*
	Yaigoje Apaporis	Cosmetidae	*Sibambeacincta*
Regional Natural Park (RNP)	Los Besotes	Cosmetidae	*Eucynortaquadripustulata*
	Serrania del Perija	Cosmetidae	*Eulibitiavictoriae*
	Sisavita	Cosmetidae	*Eulibitiahelena*
Civil Society Nature Reserve (CSNR)	La Palmita	Cosmetidae	*Eulibitiachacuamarei*
National Protective Forest Reserve (NPFR)	Bosque Oriental de Bogotá	Cosmetidae	*Libitiabipunctata*
			*Libitiacordata*
			*Rhaucusquinquelineatus*
			*Rhaucusvulneratus*
		Fissiphalliidae	*Fissiphalliussturmi*
			*Fissiphalliussympatricus*
		Stygnidae	*Phareusraptator*
	Cuenca alta del Río Cali	Cranaidae	*Holocranauscalus*
	Rio Meléndez	Cranaidae	*Holocranauslongipes*
	Río Nare	Cranaidae	*Holocranauscalcar*
	Rios Blanco y Negro	Cosmetidae	*Libitiabipunctata*
		Stygnidae	*Phareusraptator*
Regional Protective Forest Reserve (RPFR)	Laguna de pantano redondo y el nacimiento Río Susagua	Cosmetidae	*Libitiabipunctata*
	Montes de Oca	Cosmetidae	*Eucynortaquadripustulata*
Flora and Fauna Sanctuary (FFS)	Guanentá-alto río Fonce	Cosmetidae	*Eulibitiaclytemnestra*
	Iguaque	Cosmetidae	*Eulibitiamaculata*
			*Libitiaiguaque*
		Stygnidae	*Jabbastygnushuttorum*

In the species list, we utilize two types of dashes: the n-dash (–), used to indicate the reference of the original description, and the m-dash (—) to introduce each new subsequent bibliographic reference following the original description. After each species name, the m-dash is placed to separate a new citation.

Natural protected areas abbreviations: Civil Society Nature Reserve (**CSNR**); Flora and Fauna Sanctuary (**FFS**); National Protective Forest Reserve (**NPFR**); Regional Natural Park (**RNP**); Regional Protective Forest Reserve (**RPFR**); National Natural Parks (**NNP**); Regional Integrated Management Districts (**RDIM**).

## ﻿Results

### ﻿Species descriptions

**Order Opiliones Sundevall, 1833**

**Suborder Laniatores Thorell, 1876**

#### ﻿Family Cranaidae Roewer, 1913

##### ﻿ Phalangodus

Taxon classificationAnimaliaOpilionesCranaidae

Gervais, 1842

56E54EE2-952A-5FB8-967F-D1E2C341F6D0

###### Included species.

*Phalangodusanacosmetus* Gervais, 1842 (type species); *Phalangodusbriareos* Villarreal & García, 2016; *Phalangodusandresi* sp. nov.; *Phalangoduscottus* Villarreal & García, 2016; *Phalangodusgyes* Villarreal & García, 2016; *Phalangoduskuryi* Villarreal & García, 2016; *Phalangoduspalpiconus* (Roewer, 1943).

###### Diagnosis.

See Villarreal and García (2016).

##### ﻿ Phalangodus andresi sp. nov.

Taxon classificationAnimaliaOpilionesCranaidae

F4102D81-CBD0-5AB5-8DDD-A6E2F419A8FB

https://zoobank.org/B451F03A-F855-4F5B-8E90-8BDAF7890A11

Figs 1 , 2 , 3 , 4

###### Material examined.

• ***Holotype***: ♂ (ICN-Ao-1908), Colombia, Cundinamarca, San Antonio del Tequendama, R.N. Los Tunos (4.562234, -74.314527); 2,250 m; 3 Jun. 2018; (A. García, S. Galvis leg.). • ***Paratypes***: • 3 ♀♀ (ICN-Ao-1909, with one used for description), same data as the holotype; • 2 ♂♂, 1 ♀ (ICN-Ao-1003), Colombia, Cundinamarca, San Antonio del Tequendama, R.N. Los Tunos; 28 Aug. 2006; (F. Borrero leg.).

###### Diagnosis.

**Figure 1.**
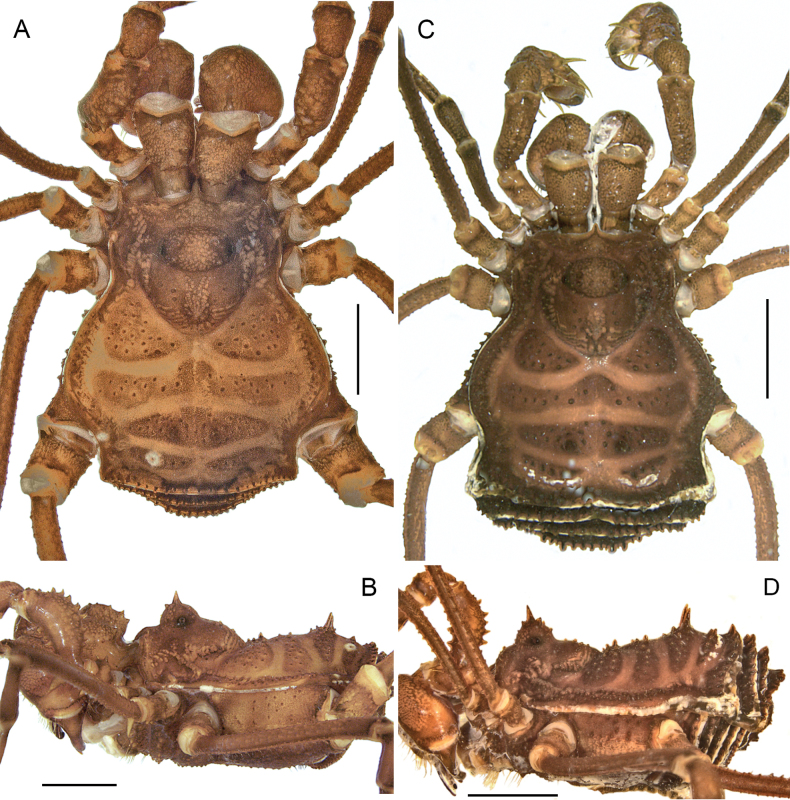
*Phalangodusandresi* sp. nov. Male holotype (ICN-Ao-1908) **A** habitus, dorsal view **B** lateral view. Female paratype (ICN-Ao-1909): **C** dorsal view **D** lateral view. Scale bars: 3 mm.

**Figure 2.**
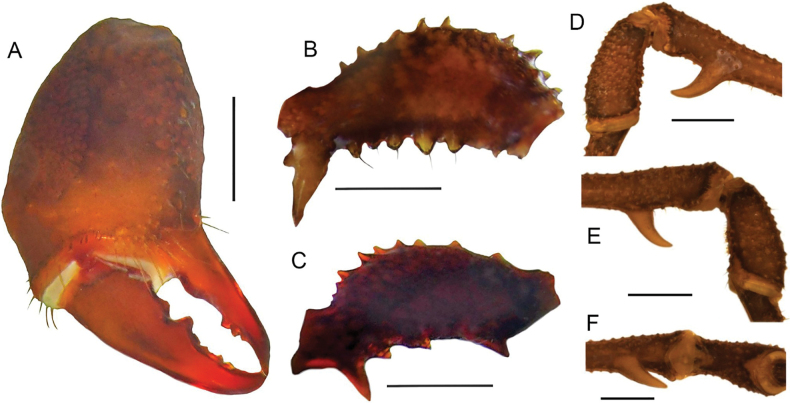
*Phalangodusandresi* sp. nov. Male holotype (ICN-Ao-1908) **A** chelicera, frontal view **B** right palp, femur, ectal view **D** right leg IV: femur distal portion and patella, prolateral view **E** ditto, retrolateral view **F** ditto, ventral view **C** female, paratype (ICN-Ao-1909), right palp, femur, ectal view. Scale bars: 1 mm.

**Figure 3.**
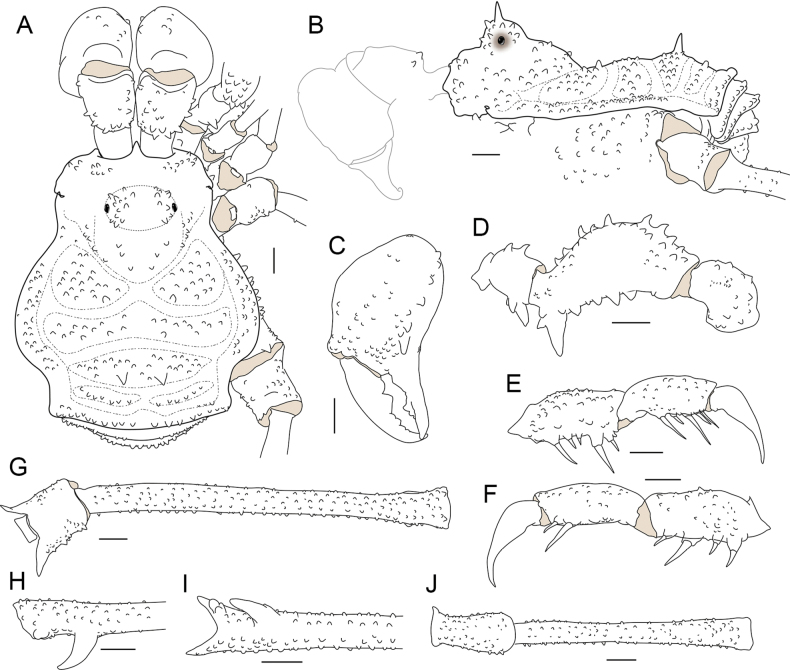
*Phalangodusandresi* sp. nov. Male holotype (ICN-Ao-1908) **A** habitus, dorsal view **B** habitus, lateral view **C** chelicera, frontal view **D** right pedipalp: trochanter, femur, and patella, ectal view **E** ditto, tibia, tarsus and claw, dorsoectal view **F** ditto, mesal view **G** right leg IV, trochanter and femur, dorsal view **H** ditto, femur, distal portion, prolateral view **I** ditto, ventral view **J** right leg IV, patella and tibia, ventral view. Scale bars: 1 mm.

*Phalangodusandresi* sp. nov. can be distinguished from all other species of the genus except *P.palpiconus* by the (1) presence of conspicuous granulation of mesotergal areas I –IV, lateral borders of dorsal scutum, ocularium and posterior region of the carapace (Figs 1A, B, 3A, B); (2) small size (males DSL ~ 10.7–11.9 mm), except *P.kuryi* (9.0–11.4 mm). It can be distinguished from the latter species by the ornamentation of the pedipalpal femur (with a very large ventroproximal tubercle absent in *P.kuryi*) (Fig. 3D), and the presence of ornamentation in leg IV of the males (Fig. 3G–J) (absent in *P.kuryi*), as well as the interocular distance, height of the ocularium and presence of paired tubercles near each eye (absent in *P.kuryi*). From *P.palpiconus*, the most morphologically similar species, it is distinguished by the ornamentation of the femur IV of the males, having a subdistal large and curved spine and a short distal bifid tubercle (Figs 2E, F, 3H, I) on the prolateral face (absent in *P.palpiconus*); lack of a retrolateral subdistal spine in the same segment (present in *P.palpiconus* (Hara et al. 2014: figs 4, 5)); the shape of the VP of the penis, more elongated and with more marked medial constrictions, and the more basal position of MS-A/B groups.

**Figure 4.**
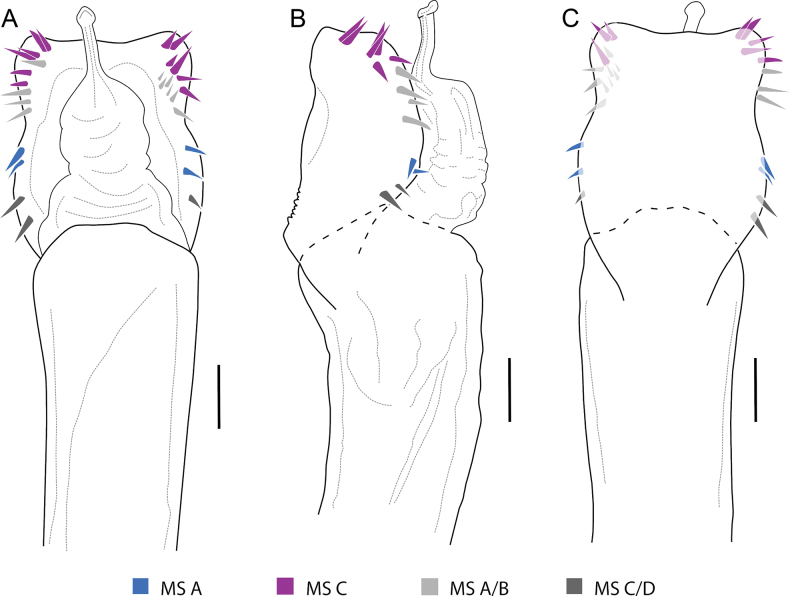
*Phalangodusandresi* sp. nov. Male paratype (ICN-Ao-1003). Penis, apical portion **A** dorsal view **B** lateral view **C** ventral view. Scale bars: 0.25 mm.

**Figure 5.**
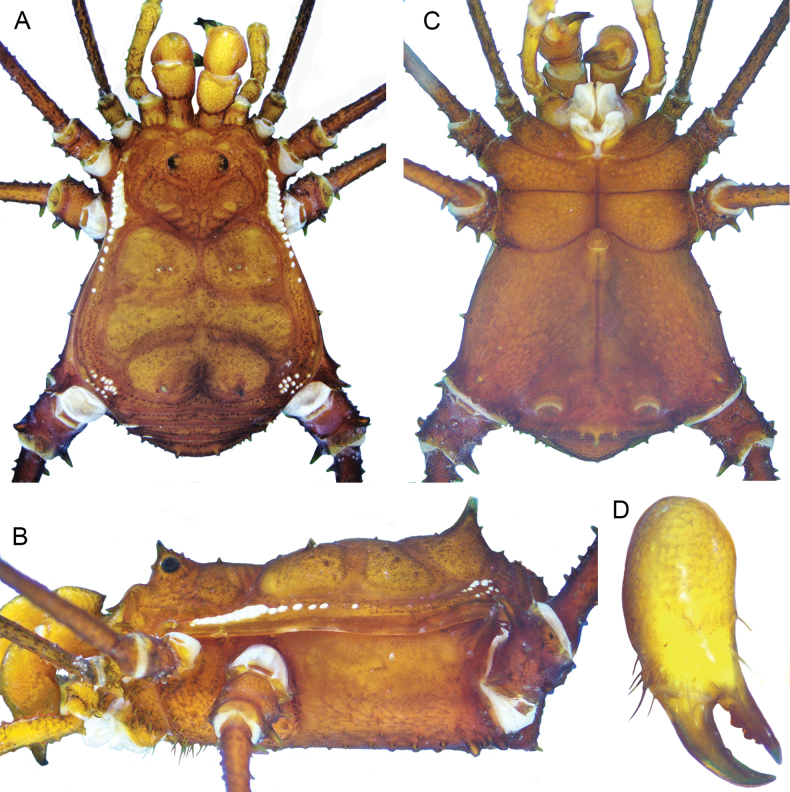
*Ventrifurcaphallaina* sp. nov. Male holotype (CIUQ-020631) **A** dorsal view **B** lateral view **C** lateral view **D** right chelicera, frontal view. Scale bars: 1 mm.

###### Description.

**Measurements of body and appendages.** Holotype ♂ (ICN-Ao-1908): DSL = 10.8; CL = 4.5; AL = 6.3; CW = 5.6; AW = 7.4; IOD = 1.6; BaCh = 2.7; FePp = 2.1, PaPp = 1.1, TiPp = 1.6, TaPp = 1.3, ClPp = 1.1; FeL I: 6.0; FeL II = 11.4; FeL III = 8.9; FeL IV = 14.1; TiL I = 4.6; TiL II = 11.3; TiL III = 5.3; TiL IV = 8.2. • Paratypes: • (ICN-AO-1909, ICN-AO-1003, 4 ♀♀, min–max): DSL = 8.3–9.3; CL = 2.9–4.5; AL = 4.0–7.8; CW = 4.9–5.8; AW = 7.2–7.9; IOD = 1.4–2.0; BaCh = 1.4–1.9; FePp = 1.9, PaPp = 1.1, TiPp = 1.4, TaPp = 1.1, ClPp = 1.0; FeL I = 2.9–3.6; FeL II = 8.1–10.6; FeL III = 7.3–9.4; FeL IV = 8.9–14.4; TiL I = 3.8–4.5; TiL II = 7.9–9.5; TiL III = 4.9–5.7; TiL IV = 5.9–9.0. • (ICN-AO-1003; 2 ♂♂, min–max): DSL = 10.7–11.8; CL = 4.9–5.3; AL = 6.6–7.4; CW = 5.3–6.3; AW = 7.4–8.8; IOD = 1.9–2.1; BaCh = 2.6–3.5; FePp = 1.9–2.1, PaPp = 1.0–1.2, TiPp = 1.5–1.7, TaPp = 1.2–1.4, ClPp = 1.1; FeL I = 5.1–6.4; FeL II = 11.1–11.1; FeL III = 9.4–10.6; FeL IV = 12.2–16.8; TiL I = 4.3–5.0; TiL II = 10.2–10.7; TiL III = 5.6–9.3; TiL IV = 8.4–10.2.

**Male holotype (ICN-Ao-1908). *Dorsum*** (Figs 1A, B, 3A, B). DS outline type alpha, with bulge longitudinally asymmetric widest at scutal groove II, lateral borders with granules only on the middle region. Carapace with few granules on the anterolateral and posterior region. Ocularium high, without median depression, with a paramedian pair of sharp tubercles and granules close to the eyes. Integumentary dome of ozopore raised and conspicuous. Abdominal scutum well delimited, divided into four well-marked scutal areas; I divided into left and right halves by invasion of the scutal area II; I and II granulated, with a pair of conspicuous medial tubercles, one tubercle on each side; III with a pair of paramedian acuminate spines and densely granulated; IV divided, with a row of six or seven granules on each side. Posterior border of the DS slightly convex and with a row of granules. Free tergites I–III with a row of granules.

***Venter*.** Coxa I with a row of large tubercles of different sizes; II longer than I and III, with two median rows of low tubercles, the anterior one more conspicuous; III densely covered with irregular rows of small tubercles and with the posterior border sigmoid; IV strongly backward, with a median row of tubercles in the medium area and lateral border, and small tubercles densely distributed. Stigmatic area with a row of small tubercles on posterior border and minute granules sparsely distributed. Stigmata large, oval, and oblique. Free sternites with a row of small granules.

***Chelicerae*** (Figs 2A, 3C). Segment I with well-defined bulla, with dorsomesal tubercles and a row of three large tubercles in the ectal region. Segment II swollen. Fixed finger with a proximal narrow and low tooth, a large gap and three subdistal median teeth. Movable finger with a proximal wide and low tooth, one large tooth and the distal inner surface irregularly dentate. Mesal side of the base of the fixed finger and near the base of the movable finger with setiferous tubercles of different sizes.

***Pedipalps*** (Figs 2B, C, 3D–F). Trochanter with a dorsal pair of paramedian tubercles. Ventrally with large bifid tubercle in distal portion. Femur slightly compressed, dorsally curved, and ventrally straight in lateral view, with dorsoectal distal row of granules, one dorsal, and one ventral row of large forward projected tubercles (the apicalmost of the ventral row bifid and thicker than the remaining), the ventrodistal portion unarmed. Mesal and ectal faces without large tubercles. Patella short, cylindrical, and curved, with a dorsal row of short tubercles and small dorsodistal granules. Tibia dorsally granulated; tibia mesal IiIi (3>1>2=4), ectal IiiIi (4>1>5>2>3). Tarsus dorsally granulated, tibia with spines only in the distal portion, mesal Ii, ectal IiIiI (3>1>5>4>2). Claw proximally swollen.

***Legs*** (Figs 1A, 2D–F, 3B, 3G–J). Coxa I and III smooth; II with one dorsal tubercle; IV with one dorsodistal domed large tubercle, and a row of large tubercles in the prolateral border. Trochanters I–III unarmed; II with a prolateral row of small tubercles; III with one retrodistal tubercle and one group of small prolateral tubercles; IV with small prolateral granules sparsely distributed and one row of small tubercles in the retrolateral border. Femora I–III straight and with longitudinal rows of granules; IV sub-straight, densely granulated, ventrally with one large subdistal tubercle hook-like shaped and one distal rounded trifid tubercle. Ratio Fe IV/DSL = 1.85. Patellae I–IV granulated. Tibiae I–V straight and densely granulated, unarmed. Metatarsi I to IV with rows of small granules, I–III unarmed, IV with a ventro-distal border pair of spines. Tarsi III and IV with subparallel smooth claws and tarsal process. Tarsal counts: 8(3)-8(3), 13(3)-14(3), 8-8, 8-7.

***Penis*** (Fig. 4). VP subsquare, with proximal and distal portion delimited by a medial constriction, and lateral rounded lobes in the proximal portion; distal border slightly concave. Glans + Stylus columnar, glans with some folds at the base, stylus normally thickened, shorter than the glans, substraight; stylar caps ring-shaped. MS-A/B groups indistinguishable from each other, located on the proximal portion of the VP, with an increase in setae and composed of three to four pairs; MS-C/D groups located in the distal portion of the VP, separated from MS-A/B by a small gap, the setae rearranged into two irregular rows, one laterodistal and one mesodorsal. MS-E presumably present, since they are very small (as in other members of the genus), and were not observable under the magnification used.

***Coloration (in alcohol)*** (Figs 1, 2). Carapace, DS borders, posterior region and ocularium reticulated in Moderate brown 58, on background Light yellowish brown 76 (in females, it is Dark brown 59 on background Light brown 57). Abdominal scutum Light yellowish brown 76 (in females, it is moderate orange 53), with the scutal areas Moderate brown 58 (in females, it is Strong brown 55). Free tergites Dark grayish yellowish brown 81. Pedipalps, chelicerae and trochanters Strong brown 55 reticulated; remaining segment of the legs Strong brown 55 reticulated with fine mottled Deep orange 51. Stigmatic area Strong brown 55. Tip of cheliceral teeth Deep reddish brown 41.

**Female (ICN-Ao-1909)** (Fig. 1C, D). Differing from male by: ocularium slightly narrower; carapace shorter; coda wider (DS outline type alpha-keyhole); tubercles of area III slightly higher. Chelicerae non-hypertelic, with movable finger thinner. Pedipalpal femur lower and thinner in lateral view. Stigmatic area shorter. Genital operculum wider. Trochanters III and IV narrower; femur IV thinner, without large ventral-subdistal tubercle with hook-like shape and without a ventrodistal rounded trifid tubercle. For color differences, see the color description of the male.

***Ovipositor*.** Dorsal lobes (dl) and ventral lobes (vl) rounded, with four and two pairs of large, acuminated, single-tipped setae, respectively. The dl with three pairs of dorsal setae (ds) distally located and one pair basally located. Lateral region of the ovipositor with one pair of dorso-lateral setae (dls).

###### Distribution.

Known only from the type locality.

###### Etymology.

The species is named in honor of our colleague and friend, the arachnologist Andrés F. García, who has greatly enriched the field’s knowledge of Opiliones in Colombia and described the vast majority of species within the genus *Phalangodus*; moreover, he was the collector of the type series for this species.

##### ﻿ Ventrifurca

Taxon classificationAnimaliaOpilionesCranaidae

Roewer, 1913

1A326DFD-C42F-5BA9-AD54-B23750D8F965

###### Included species.

*Ventrifurcaabnormis* (Roewer, 1932); *Ventrifurcaalbipustulata* Roewer, 1913 (type species); *Ventrifurcacaffeinica* Villarreal, Kury & Pinto-da-Rocha, 2015; *Ventrifurcadybasi* (Goodnight & Goodnight, 1947); *Ventrifurcaphallaina* sp. nov.

###### Diagnosis.

Villarreal et al. (2015).

##### ﻿ Ventrifurca phallaina sp. nov.

Taxon classificationAnimaliaOpilionesCranaidae

A53E326C-59B4-5679-BB2A-BCC283D29198

https://zoobank.org/8101A255-1326-46E3-91C7-5D564C59FFC1

**Figure 6.**
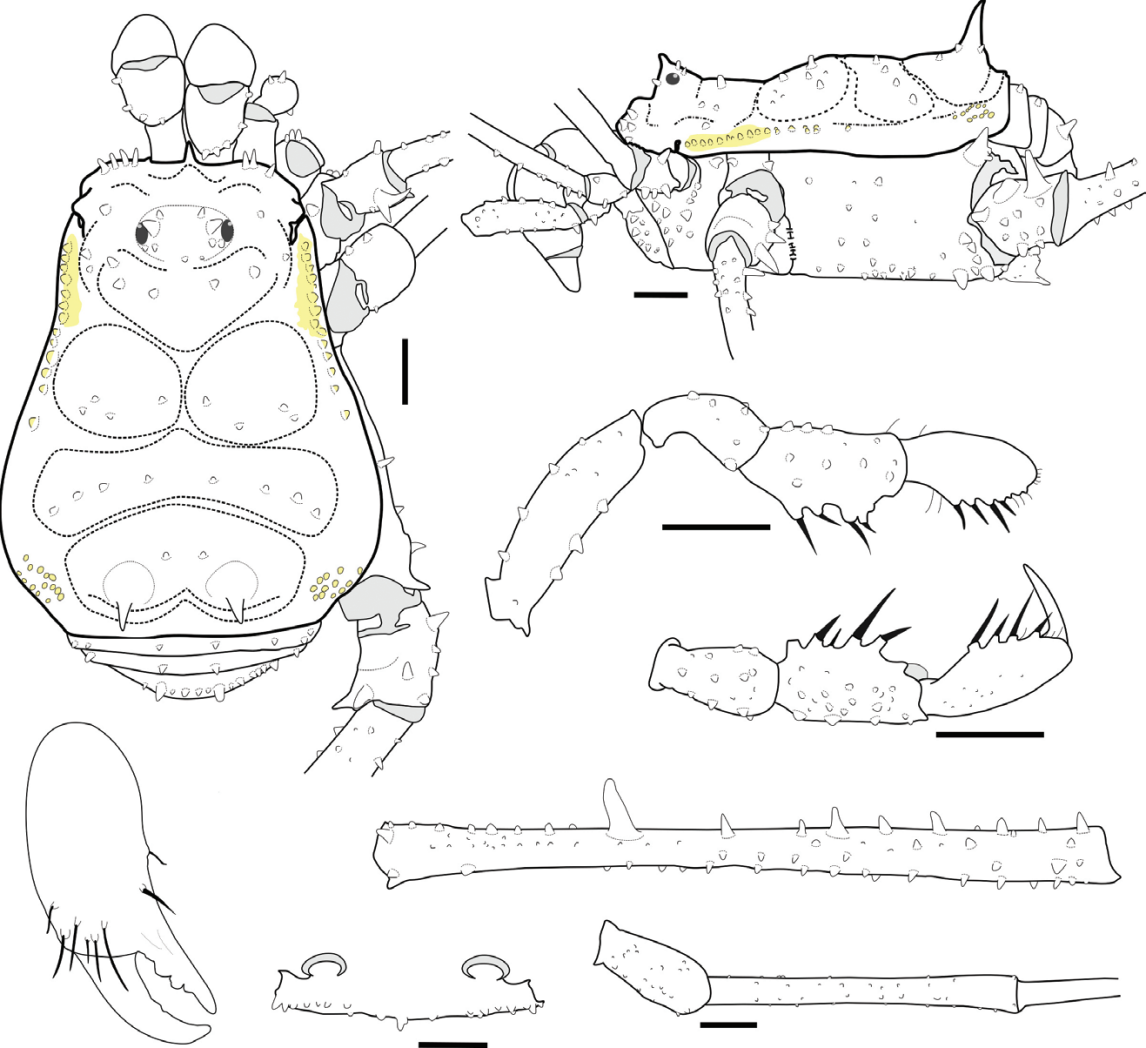
*Ventrifurcaphallaina* sp. nov., male paratype (MIZA 0105869) **A** habitus, dorsal view **B** habitus, lateral view **C** stigmatic area, posterior portion, ventral view **D** chelicera, frontal view **E** right pedipalp, ectal view **F** ditto, mesal view **G** right leg: femur, dorsal view **H** ditto, patella and tibia, dorsal view. Scale bars: 1 mm.

**Figure 7.**
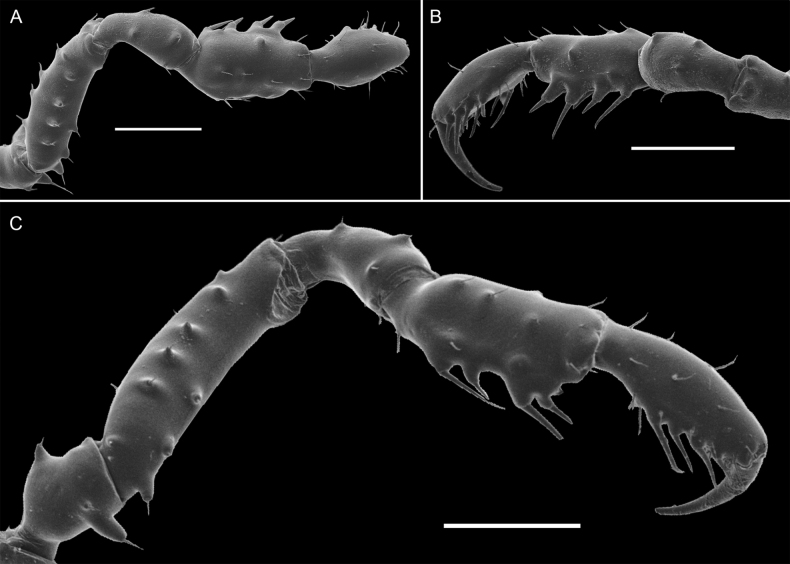
*Ventrifurcaphallaina* sp. nov., male paratype (MIZA 0105869), right pedipalp **A** dorsoectal view **B** patella, tibia, tarsus, and claw, dorsomesal view **C** ectal view. Scale bars: 1 mm.

**Figure 8.**
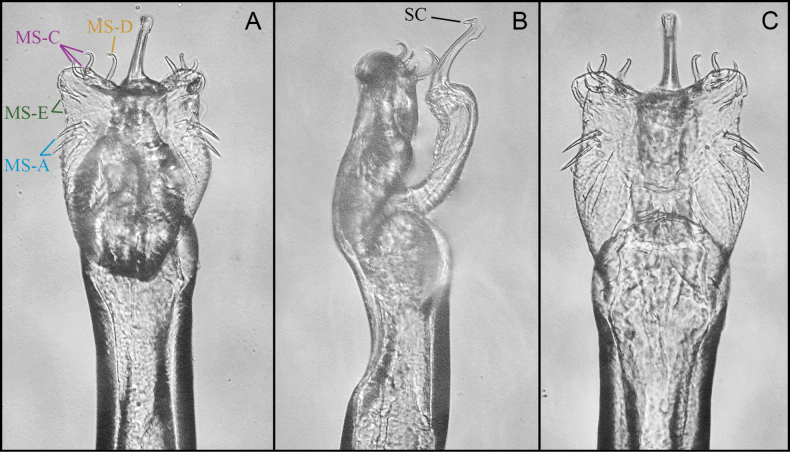
*Ventrifurcaphallaina* sp. nov., male paratype (CIUQ-020633), penis **A** dorsal view **B** lateral view **C** ventral view. Abbreviations: MS = macrosetae of penis; MS-A = proximal group of setae; MS-B ventroproximal group of setae; MS-C = distal group of setae; MS-D = dorso medial group of setae; MS-E = ventrodistal group of reduced setae, SC = stylar caps. Figures not scaled.

**Figure 9.**
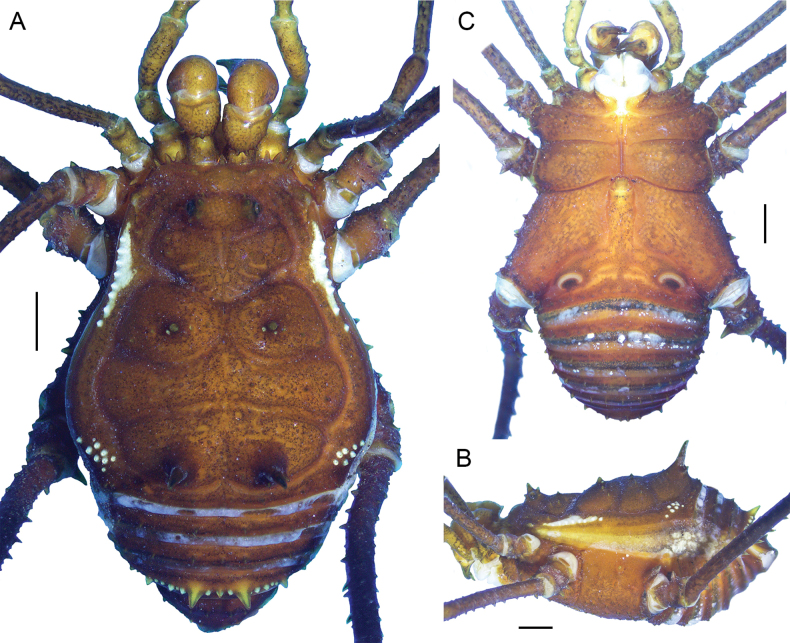
*Ventrifurcaphallaina* sp. nov., female paratype (CIUQ-020632) **A** dorsal view **B** lateral view **C** ventral view. Scale bars: 1 mm.

Figs 5 , 6 , 7 , 8 , 9

###### Material examined.

• ***Holotype***: ♂ (CIUQ-020631), Colombia – Valle del Cauca, Buenaventura, (3.8375, -77.2436); 56 m; 12 Nov. 2022; (A. L. García, L. Delgado-Santa leg.). • ***Paratypes***: • 1 ♀ (CIUQ-020632), same data as the holotype • 1 ♂ (MIZA 0105869) same data as the holotype • 1 ♂ (MNRJ 1596) same data as the holotype. ***Other material examined*.** • 3 ♂♂, 1 ♀ (CIUQ-020634) same data as the holotype.

###### Diagnosis.

*Ventrifurcaphallaina* sp. nov. can be distinguished from *V.abnormis* by the scarce ornamentation of the mesotergal areas I to III; also, can be distinguished from all other species of the genus by the ornamentation pattern of the yellow tubercles on dorsal scutum, restricted to the lateral of carapace and posterolateral portion of DS (Figs 5A, B, 6A, B, 9A, B); the presence of a yellow spot on the anterolateral portion of the carapace; the shape and size of posteroventral projections on the stigmatic area, simple and short (Figs 5C, 6C), instead of bifid and globular (*V.albipustulata*) or large and digitiform or curve (*V.caffeinica* and *V.abnormis*); and the absence of yellow tubercles on the mesotergal area III, behind the paired spines (present in *V.albipustulata* and *V.caffeinica*). The genital morphology is very similar to *V.albipustulata*, with slight differences in relation to the shape of the concavity of the truncus (Fig. 8).

###### Description.

***Measurements of body and appendages*.** Holotype: (CIUQ-020631 ♂). DSL = 8.3, CL = 3.1, AL:5.2, CW = 3.2, AW = 5.3, IOD = 0.9; BaCh = 0.8, FePp = 2.1, PaPp = 1.1, TiPp = 1.5, TaPp = 1.3, ClPp = 0.9; FeL I = 3.8, FeL II = 9.8, FeL III = 7.6, FeL IV = 9.8, TiL I = 2.8, TiL II = 7.4, TiL III = 3.0, TiL IV = 5.4. Paratypes: • (MNRJ 1596, 1 ♂; MIZA 0105869 1 ♂): DSL = 7.8–8.4; CL = 3.4–3.2, AL = 3.9–5.3, CW = 4.0–4.3, AW = 6.5–6.7, IOD = 1.1–1.2; BaCh = 0.8–0.9; FePp = 2,3, PaPp = 1.3, TiPp = 1.7, TaPp = 1.2; ClPp = 1.3; FeL I = 4.6–4.8, FeL II = 10.6–10.8, FeL III = 7.4–8.1, FeL IV = 10.7–11.2, TiL I = 3.1–3.3, TiL II = 7.9–8.9, TiL III = 3.7–4.3, TiL IV = 5.9–6.0. • (CIUQ-020632, ♀): DSL = 6.0, CL = 2.7, AL = 2.9, CW = 3.0, AW = 5.2, IOD = 1.1; BaCh = 0.9; FePp = 2.2, PaPp = 1.2, TiPp = 1.6, TaPp = 1.1, ClPp = 1.1; FeL I = 2.6, FeL = II, 7.4, FeL III = 6.2; FeL IV = 8.1, TiL I = 1.5, TiL II = 5.2, TiL III = 3.5, TiL IV = 4.6.

**Male holotype (CIUQ-020631). *Dorsum*** (Figs 5A, B, 6A, B). DS outline type Gamma pyriform. Mesotergum widest at groove III level; lateral margin of DS with yellow rounded tubercles from carapace to area I, following outline of scutum. Carapace with three or four tubercles on each side of the anterior border. Ocularium low, armed with a paramedian pair of acuminate forward inclined tubercles and some small tubercles near the eyes. Integumentary dome of ozopore raised and conspicuous. Mesotergum well delimited, divided into three well-marked areas: area I divided into two roughly ellipsoidal halves by longitudinal groove, with a paramedian pair of large tubercles and four small tubercles; II penetrating subtly into I, with a median transverse row of eight small tubercles; III with a pair of paramedian acuminate high subparallel spines, located near the posterior margin; vestigial groove between III and IV present, partially blurred and located just behind the spines. Posterior border of DS straight. Free tergites I–III with a pair of paramedian large granules, I to II with one to three smaller laterodistal granules on each side; III armed with a posterior row of 11 tubercles, the paramedian pair much stouter.

***Venter*** (Figs 5B, C, 6B, C). Coxa I with a row of tubercles of different size; coxa II longer than coxae I and III, with a cluster of distal tubercles of different size; coxa IV strongly backward. Stigmatic area with minute granules sparsely distributed, posterior border with posterodistal process very small. Stigmata large and oval.

***Chelicerae*** (Figs 5D, 6D). Segment I with well-defined bulla, with a row of four or five tubercles in ectal region. Segment II slightly swollen, with a row of large setiferous tubercles near to the base of the movable finger and a few mesal setiferous tubercles. Fixed finger with three medial teeth, decreasing in size. Movable finger with one large and wide medial tooth, accompanied by two flat, low teeth on each side.

***Pedipalps*** (Figs 6E, F, 7). Trochanter with tubercles, in dorsal and ventral region. Femur cylindrical, slightly elongated, armed with a longitudinal row of four tubercles in dorsal side and three tubercles in ventral side. Patella short, cylindrical, and curved, with row of tubercles in dorsal view, one ectodistal and two parallel rows of tubercles in mesal view tubercles.

***Legs*** (Figs 5A–C, 6A, B, G, H). Coxae I and II with one anterior row of large tubercles; III with two rows and IV with two diagonal rows. Trochanter I with two prolateral, two retrolateral and four ventral tubercles; II with two prolateral, two retrolateral and five ventral tubercles; III with four prolateral, two retrolateral and four ventral tubercles; IV with one prolateral, one retrolateral and five ventral tubercles, more conspicuous than those from the other trochanters. Tubercles of trochanter IV more conspicuous than the others. Femora I to III straight, with longitudinal rows of granules; IV curved in its proximal portion, with seven longitudinal rows of tubercles, the retrolateral row with large proximal tubercles, and one large subdistal tubercle on the same row. Patellae I–IV with small tubercles. Tibiae and metatarsi I–V straight and granulate, unarmed. Claws III and IV smooth, with two minute proximal ventral protuberances. Ratio Fe IV/DSL = 1.35. Tarsal process present. Tarsal counts 7(3)-7(3), 14(3)-14(3), 7-7, 7-7.

***Penis*** (Fig. 8). Ventral plate (VP) with concave lateral margins, with subdistal constriction and the distal corners ear-shaped, apical wide u-shaped cleft. Glans + Stylus complex surpassing the VP, glans columnar, with folds at the base, stylus normally thickened, sub-straight; stylar caps foliar-shaped, short. MS-A1–A2 straight, located at the distal portion of the VP; MS-B absent; MS-C1–C3 curve, close to each other, and located in the distal part of the VP; MS-D1 larger than MS-C group, curve and located medially to MS-C, MS-D2 absent; MS-E1–E2 small, located on the flange of the VP. Truncus with ventral concavity next to the VP.

***Coloration (in alcohol)*** (Fig. 5). Carapace, border of the DS, scutal grooves and adjacencies, and free tergites reticulated Dark brown 59, on background Brownish orange 54 and Brilliant orange yellow 67. Scutal areas Brilliant yellow 83. Trochanters I–III same as the carapace; IV Blackish red 21 and Brownish black 65. Pedipalps and chelicerae strong yellow 84 reticulated. Stigmatic area Strong yellowish brown 74. Tip of cheliceral teeth Dark olive 108.

**Female (CIUQ-020632)** (Fig. 9). Differing from male by carapace shorter than male, coda wider, femur IV thinner and with less conspicuous spination, free tergite spines larger, and stigmatic area without posterior process.

###### Distribution.

Known only from the type locality.

###### Etymology.

*Phallaina* is a Greek word that means whale. It is used in apposition as a reference to the humpbacked shape of the species in lateral view.

#### ﻿Family Ampycidae Kury, 2003

##### ﻿ Ampycella

Taxon classificationAnimaliaOpilionesAmpycidae

Roewer, 1929

2D7612C7-8A42-5544-802F-CB83DC574CE2

###### Included species.

*Ampycellafrizzellae* (Mello-Leitão, 1942); *Ampycellaspiniventris* Roewer, 1929 (type species); *Ampycellafortunata* sp. nov.

###### Diagnosis.

**Figure 10.**
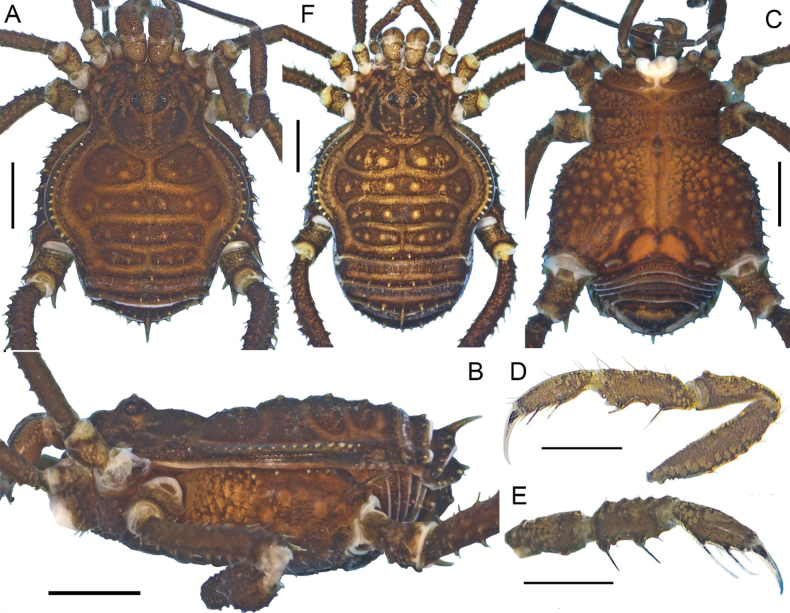
*Ampycellafortunata* sp. nov., male holotype (CIUQ-020635) **A** dorsal view **B** lateral view **C** ventral view **D** right pedipalp: mesal view **E** ditto, ectal view. Female paratype (CIUQ-020636): **F** dorsal view. Scale bars: 1 mm.

**Figure 11.**
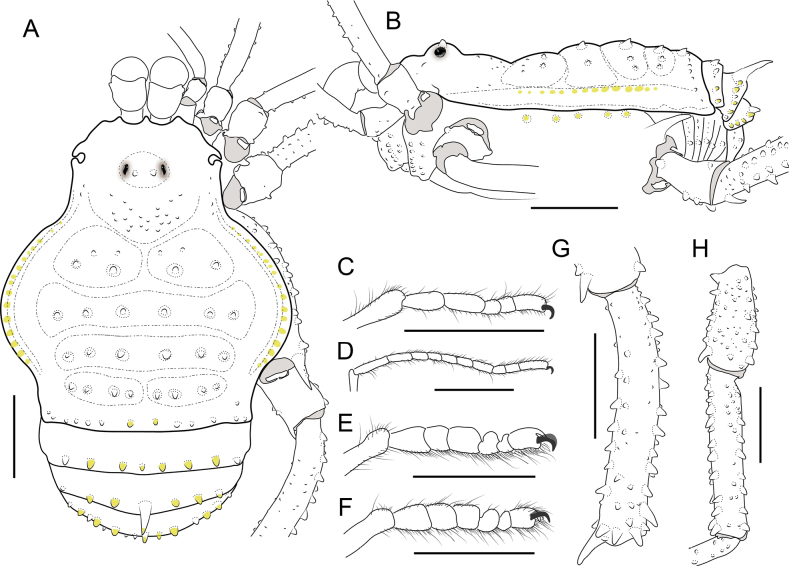
*Ampycellafortunata* sp. nov., male holotype (CIUQ-020635) **A** habitus, dorsal view **B** habitus, lateral view **C–F** tarsus I–IV, retrolateral view **G** right trochanter and femur IV, dorsal view **H** patella and tibia IV, dorsal view. Scale bars: 1 mm.

**Figure 12.**
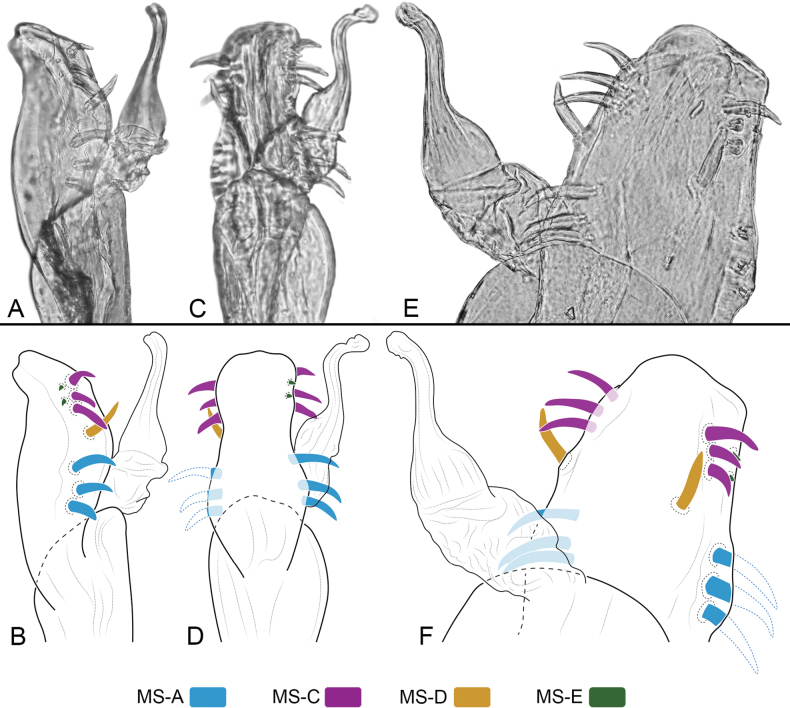
*Ampycellafortunata* sp. nov., male paratype (CIUQ-020637), penis **A, B** lateral view **C, D** ventral view **E, F** dorsolateral view. Figures not scaled.

Outline of DS type alpha; four mesotergal areas well defined and unarmed; DS with small yellowish tubercle on the lateral margins (Fig. 10A, B, F). Free tergites II and III with a posteromedial spine (Fig. 11B). Penis (only known to *A.fortunata* sp. nov.): VP sub-rectangular with medial constriction, giving it a guitar-shaped appearance, and distal border without cleft; MS-A1–A3 in the medial portion of the VP; MS-B absent; MS-C1–C3 distally located, similar to each other; MS-D1 as large as MS-A similar to each other, medially located and pointing distally. Stylus clearly differentiated from the glans, slightly curved in the distal portion (Fig. 12).

##### ﻿ Ampycella fortunata sp. nov.

Taxon classificationAnimaliaOpilionesAmpycidae

0B6A1434-DA4F-5613-8D49-98B0910DCA81

https://zoobank.org/89231F4C-833A-4627-A866-2F33D9849AE0

Figs 10 , 11 , 12

###### Material examined.

• ***Holotype***: ♂ (CIUQ-020635), Colombia – Valle del Cauca, Buenaventura, (3.8375, -77.2436); 56 m; 12 Nov. 2022; (A. L. García, L. Delgado-Santa leg.). • ***Paratypes***: • 1 ♀ (CIUQ-020636), same data as the holotype • 2 ♂♂ (CIUQ-020637; CIUQ-020638) same data as the holotype.

###### Diagnosis.

*Ampycellafortunata* sp. nov. can be distinguished from the other two species of the genus by the size ratio between the spines on the free tergites: spine on free tergite II larger than the spine on free tergite III (Figs 10A, B, F, 11A, B) (vs III larger than II). Additionally, it can be distinguished from *A.frizzellae* by having a low ocularium with two small tubercles (vs high ocularium with two erect spines) and the entire scutal area II (vs divided into three lobes); and from *A.spiniventris* for having the anterolateral part of scutal area II projected forwards, anterior to the middle part (vs anterolateral parts similar to the medial portion), and having longer dorsal spines on the coxa IV.

###### Description.

***Measurements of body and appendages*.** Holotype: (CIUQ-020635) DSL = 3.5, CL = 1.5, AL = 1.6, CW = 1.6, AW = 3.2, IOD = 0.4, BaCh = 0.5, FePp = 1.1; PaPp = 0.6, TiPp = 0.6, TaPp = 0.5, ClPp = 0.3, FeL I = 1.7, FeL II = 2.8, FeL III = 2.2, FeL IV = 2.4, TiL I = 1.3, TiL II = 2.2, TiL III = 1.4, TiL IV = 1.4. Paratype: (CIUQ-020636 ♀) DSL = 3.5, CL = 1.4, AL = 2.0, CW = 1.7, AW = 3.4, IOD = 0.4, BaCh = 0.5, FePp = 1.1; PaPp = 0.6, TiPp = 0.7, TaPp = 0.5; ClPp = 0.3, Fe I = 1.4, FeL II = 2.7, FeL III = 2.1, FeL IV = 2.4, TiL I = 1.2, TiL II = 2.2, TiL III = 1.3, TiL IV = 1.5. Paratypes: (CIUQ-020637; CIUQ-020638, 2 ♂♂, min–max) DSL = 3.3–3.4, CL = 1.4–1.4, AL = 1.6–1.6, CW = 1.6–1.6, AW = 3.2–3.2, IOD = 0.4–0.4, BaCh = 0.4–0.4, FePp = 1.1–1.1; PaPp = 0.5–0.6, TiPp = 0.5–0.6, TaPp = 0.5–0.6, ClPp = 0.3–0.3, FeL I = 1.3–1.4, FeL II = 2.5–2.6, FeL III = 2.1–2.1, FeL IV = 2.2–2.3, TiL I = 1.1–1.1, TiL II = 2.1–2.1, TiL III = 1.2–1.3, TiL IV = 1.3–1.4.

**Male holotype (CIUQ-020635). *Dorsum*** (Figs 10A, B, 11A, B). DS outline type alpha. Dorsal scutum widest at scutal area II; lateral borders of dorsal scutum with yellowish granules only on the middle region. Carapace mostly smooth, with a group of granules on the posterior region. Ocularium located slightly posterior to the ozopores, with a paramedian pair of small tubercles. Mesotergum well delimited, divided into four well-marked areas: I divided medially into left and right halves, with a medial large tubercle on each half and one small lateral tubercle and two anterior granules on each side; II and III entire, with a paramedian pair of large tubercles and two to three pairs on each side; IV divided into two halves, with four tubercles on each side. Posterior border of the DS substraight, with a row of tubercles. Free tergites I with a row of tubercles; II and III with a medial large spine (spine in II larger than in III) and some lateral tubercles, some of them yellowish colored.

***Venter*** (Figs 10B, C, 11B). Stigmatic area with the posterior border convex, unarmed, only with minute granules sparsely distributed. Stigmata large, oval, and subparallel. Coxa I with two rows of large tubercles of different sizes; coxa II longer than coxae I and III, II and III with two rows of low tubercles, the anterior ones more conspicuous; coxa IV strongly backward and widened, conspicuously wider than the anterior coxae, with large tubercles in the posterior and lateral regions. Free sternites each with a row of small tubercles, the lateral larger.

***Chelicerae*** (Fig. 11A). Chelicerae not swollen. Fixed finger with five teeth. Movable finger with ten teeth, one large tooth and distal inner surface dentate. Mesal side of the base of the fixed finger and near the base of the movable finger with setiferous tubercles of different sizes.

***Pedipalps*** (Figs 10D, E). Trochanter unarmed. Femur slightly compressed, sub straight in lateral view, unarmed. Patella with one large dorsodistal tubercle and some granules. Tibia shorter than femur, dorsally smooth; tibia mesal II, ectal IIi. Tarsus shorter than tibia, dorsally smooth; tarsus mesal IIi, ectal IiIiii.

***Legs*** (Figs 10A, 11C–H). Coxa I to III with one anterior row of small tubercles; IV with five longitudinal rows and with a very large dorsodistal spiniform tubercle. Trochanters I and II with two prolateral, two retrolateral, and three ventral tubercles; III with three prolateral, three retrolateral, and three ventral tubercles; IV with three prolateral, three retrolateral, and four ventral tubercles. Tubercles of trochanter IV are more conspicuous than the others. Femora I and II without conspicuous ornamentation; femora III and IV with seven rows of spines. Patellae I–IV with small tubercles. Tibiae I–IV straight and with tubercles; IV with a row of conspicuous tubercles. Claws III and IV smooth. Ratio Fe IV/DSL = 0.69. Tarsal counts 5(3)-5(3), 10(3)-10(3), 6-6, 6-6.

***Penis*** (Fig. 12). Ventral plate (VP) sub-rectangular with medial constriction giving it a guitar-shaped appearance; distal border straight, without distal cleft. Glans + Stylus complex columnar, both parts well differentiated; stylus normally thickened, slightly curved S-shaped. MS-A1–A3 aligned in the basal part of the VP; MS-B absent; MS-C1–C3 aligned distally, similar in size and shape; MS-D1 large, similar to MS-A, distally pointed, MS-D2 absent; MS-E1-E2 very small, near MS-C cluster.

***Coloration (in alcohol)*** (Fig. 10A–E). Carapace, chelicerae, and legs reticulated Dark yellowish brown (78) on Strong yellowish brown (74), borders of the DS and free tergites Dark yellowish brown (78). Abdominal scutum Strong yellowish brown (74), with the scutal areas Dark yellowish brown (78); Coxae dorsally reticulated on background Dark orange yellow (72). Pedipalps, reticulated. Stigmatic area with two oval areas Dark orange yellow (72) surrounded by Dark yellowish brown (78). Tarsomeri III and IV Grayish olive (110).

**Female (CIUQ-020636)** (Fig. 10F). Distinguished from male by carapace broader and the coda wider, genital operculum wider, spines on coxae IV present but less conspicuous than those found in males. and distal spine of femur smaller than in males.

###### Distribution.

Known only from the type locality.

###### Etymology.

*fortunata* is a Latin word that means happy, lucky, and blessed. It is used in reference to the meaning of the name of the type locality Buenaventura.

###### Remarks.

Among the known genitalia, only *Licornustama* Villarreal & Kury, 2012 and *Hernandarioidesplana* F.O. Pickard-Cambridge, 1905 have an elongated ventral plate. However, both genera have a subtle distal cleft in the distal border of the VP, which is not the typical deep cleft seen in the subfamily (e.g., *Hexabunus* Roewer, 1913, *Hutamaia* Soares & Soares, 1977, *Thaumatocranaus* Roewer, 1932). *Ampycellafortunata* sp. nov. shares the presence of an elongated ventral plate, but it is unique in not having a distal cleft. Additionally, a pair of large, apically pointing MS-D is observed in *Ampycellafortunata*, which is also observed in some of the genera whose genitalia is known (e.g., *Hexabunus*, *Hernandarioides* Pickard-Cambridge, 1905).

### ﻿Faunistics

#### ﻿Species list recorded or described from Colombia

Only those species described, registered, or modified after 2003 are provided with a detailed logonymy here. For the remaining species, refer to the catalog by Kury (2003). When possible, we added information about the WWF ecoregion where the species is found according to available records.

#### ﻿Suborder Laniatores Thorell, 1876

**Infraorder Grassatores Kury, 2002**

**Incertae sedis**

***Cleombrotusminutus* Sørensen, 1932**

**Distribution.** Unknown department.

***Philacarussamoides* Sørensen, 1932**

**Distribution.** Unknown department. Originally cited from Ecuador, but Kury (2003) commented: “Neu Granada” (this name refers to the region of Darien Gulf, bordering Panama).

**Remarks.** Nueva Granada was a historical region of northern South America. The Viceroyalty of New Granada (1717–1723, 1739–1810, 1815–1822) was a political and administrative entity established by Spain in the 18^th^ century that encompassed a large part of the Andean region of South America, including present-day Colombia, Ecuador, Panama, and Venezuela. It was governed by a viceroy appointed by the Spanish crown. After gaining independence from Spain in 1819, the Republic of New Granada (1831–1858) was established as a sovereign state in the northern Andean region of South America, encompassing present-day mainly Colombia and Panama. The republic underwent various changes in its name and territorial boundaries.

##### Gonyleptoidea Sundevall, 1833

**Agoristenidae Šilhavý, 1973**

***Andrescavasturmi* Roewer, 1963**

**Distribution.** Huila. Magdalena Valley dry forests (NT0221).

***Avimascabra* (Roewer, 1963)**

**Distribution.** Cundinamarca. Magdalena Valley montane forests (NT0136).

***Avimatroglobia* (Pinto-da-Rocha, 1996)**

*Trinellatroglobia* Pinto-da-Rocha, 1996: 321, figs 4, 8, 15, 16. — Kury 2003: 34.

*Avimatroglobia* — Villarreal and Kury 2009: 3. — García et al. (2022): 59, figs 4, 6A.

**Distribution.** La Guajira. Cordillera Oriental montane forests (NT0118).

**Remarks.** The type locality for this species is the state of Zulia, in Venezuela. It was recorded from Colombia by García et al. (2022).

***Avimatuttifrutti* García & Pastrana-M, 2021**

*Avimatuttifrutti* García & Pastrana-M, 2021: 2, figs 1–4.

**Distribution.** Córdoba. Northern Andean montane forest (NT0145).

***Avimavenezuelica* Soares & Avram, 1981**

*Avimavenezuelica* Soares & Avram, 1981: 95. — Villarreal and Kury 2009: 67. — García et al. (2022): 60, figs 5, 6A.

*Vimavenezuelica* — González-Sponga 1987: 543, figs 708–713.

*Trinellavenezuelica* — Pinto-da-Rocha 1996: 323. — Kury 2003: 34.

**Distribution.** La Guajira. Cordillera Oriental montane forests (NT0118).

**Remarks.** The type locality for this species is the state of Zulia, in Venezuela. It was recorded from Colombia by García et al. (2022).

***Avimawayuunaiki* García, Vargas & Gutiérrez, 2022**

*Avimawayuunaiki*García et al., 2022: 56, figs 1–3, 6A, C.

**Distribution.** La Guajira. Guajira-Barranquilla xeric scrub (NT1308).

***Barinasguanenta* García & Ahumada-C., 2022**

*Barinasguanenta* García & Ahumada-C., 2022: 3, figs 1, 2, 4.

**Distribution.** Santander. Magdalena Valley montane forests (NT0136).

***Barinaspiragua* Ahumada-C. & García, 2020**

*Barinaspiragua* Ahumada-C. & García in Ahumada-C. et al., 2020: 635, figs 2–5. — García and Ahumada-C. 2022: 5.

**Distribution.** Bolívar, La Guajira, Magdalena. Magdalena-Urabá moist forests (NT0137), Guajira-Barranquilla xeric scrub (NT1308), and Amazon-Orinoco-Southern Caribbean mangroves (NT1401).

***Leptostygnusleptochirus* Mello-Leitão, 1940**

*Leptostygnusleptochirus* Mello-Leitão, 1940: 306. — Soares 1945: 383. — Soares et al. 1992: 7. — Kury 1993: 129, figs 1–6. — Flórez and Sánchez-C 1995: 369. — García and Villarreal 2020: 5, figs 1, 8, 9.

**Distribution.** Norte de Santander. Catatumbo moist forests (NT0108).

***Leptostygnusyarigui* García & Villarreal, 2020**

*Leptostygnusyarigui* García & Villarreal, 2020: 7, figs 3–6, 8, 9.

**Distribution.** Santander. Magdalena Valley montane forests (NT0136).

***Muscopilioonod* Villarreal & García, 2021**

*Muscopilioonod* Villarreal & García, 2021: 157, figs 1–10.

**Distribution.** Cundinamarca. Magdalena Valley montane forests (NT0136).

***Nemastygnusovalis* Roewer, 1929**

*Nemastygnusovalis* Roewer, 1929: 277, fig. 44. — Kury 2003: 145. — Pinto-da-Rocha et al. 2012: 30, figs 1A–C.

**Distribution.** Cundinamarca. Magdalena Valley montane forests (NT0136).

***Paravimalokura* García & Villarreal, 2023**

*Paravimalokura* García & Villarreal, 2023: 420, figs 5, 6, 18.

**Distribution.** Norte de Santander. Cordillera Oriental montane forests (NT0118).

***Paravimamagistri* García & Villarreal, 2023**

*Paravimamagistri* García & Villarreal, 2023: 424, figs 7, 8, 18, 19G.

**Distribution.** Cundinamarca. Magdalena Valley montane forests (NT0136).

***Sabanillaornata* Roewer, 1913**

**Distribution.** Atlántico. Guajira-Barranquilla xeric scrub (NT1308).

***Vimapanita* García & Kury, 2020**

*Vimapanita* García & Kury, 2020: 71, figs 5–8.

**Distribution.** Caquetá. Napo moist forests (NT0142)

##### Ampycidae Kury, 2003

***Ampycellafortunata* sp. nov.**

**Distribution.** Valle del Cauca. Chocó-Darién moist forests (NT0115).

***Ampycustelifer* (Butler, 1873)**

*Gonyleptestelifer* Butler, 1873: 116, figs 3, 4.

*Itequahyensiferus* Mello-Leitão, 1949: 29, fig. 9.

*Ampycustelifer* — Simon 1879: 241. — Roewer 1913: 49, fig. 18; 1923: 411, fig. 506. — Mello-Leitão 1923: 117; 1932: 208, fig. 119. — Goodnight and Goodnight 1943: 10. — Soares and Soares 1954: 234. — García 2014: 93, fig. 1a, b.

**Distribution.** Amazonas. Solimões-Japurá moist forests (NT0163).

**Remarks.** The type locality of this species is in the state of Amazonas, in Brazil. It was recorded for Colombia by García (2014).

***Licornustama* Villarreal & Kury, 2012**

*Licornustama* Villarreal & Kury, 2012: 73, figs 1–19. — García 2014: 93, fig. 2a, b.

**Distribution.** Meta. Apure-Villavicencio dry forests (NT0201).

**Remarks.** The type locality for this species is the state of Táchira, in Venezuela. Recorded for Colombia by García (2014).

***Thaumatocranausmagnificus* Hara, Bragagnolo & Pinto-da-Rocha, 2017**

*Thaumatocranausmagnificus*Hara et al., 2017: 463, figs 3, 4, 7D, E.

**Distribution.** Amazonas. Caqueta moist forests (NT0107).

***Thaumatocranaussplendidus* Hara, Bragagnolo & Pinto-da-Rocha, 2017**

*Thaumatocranaussplendidus*Hara et al., 2017: 466, figs 5, 6, 7F, G.

**Distribution.** Amazonas. Solimões-Japurá moist forests (NT0163).

##### Cosmetidae Koch, 1839

***Cosmetusacanti* Medrano, Kury & Martínez, 2021**

*Cosmetusacanti*Medrano et al., 2021b: 435, figs 5A–D, 6A–E, 7A–C, 11.

**Distribution.** Chocó. Chocó-Darién moist forests (NT0115).

***Cosmetusvillarreali* Medrano, Kury & Martínez, 2021**

*Cosmetusvillarreali*Medrano et al., 2021b: 439, figs 8A–D, 9A–H, 10A–C, 11.

**Distribution.** Chocó. Chocó-Darién moist forests (NT0115).

***Cynortacalcarapicalis* Roewer, 1912**

**Distribution.** Bolívar (type locality) and Cundinamarca. Sinú Valley dry forests (NT0229) and Magdalena Valley montane forests (NT0136).

***Cynortaclavipus* Roewer, 1928**

**Distribution.** Unknown department.

***Cynortaclunipecten* Roewer, 1947**

**Distribution.** Unknown department.

**Remarks.**Kury (2003) refers to the Boyacá or Santander departments as possible localities.

***Cynortadariensis* Roewer, 1925**

**Distribution.** Chocó. Chocó-Darién moist forests (NT0115).

***Cynortalateralis* Roewer, 1928**

**Distribution.** Chocó. Chocó-Darién moist forests (NT0115).

***Cynortamaculorum* Goodnight & Goodnight, 1943**

**Distribution.** Unknown department.

***Cynortarorida* Roewer, 1928**

**Distribution.** Chocó. Chocó-Darién moist forests (NT0115).

***Cynortasimplex* Roewer, 1928**

**Distribution.** Unknown department.

***Cynortellinalineata* Roewer, 1915**

**Distribution.** Tolima (without a specific locality).

***Cynortosomareticulatum* Roewer, 1947**

**Distribution.** Tolima (without a specific locality).

***Erginulusaustralis* (Roewer, 1916)**

**Distribution.** Unknown department.

***Eucynortaquadripustulata* (Simon, 1879)**

*Cynortaquadripustulata* Simon, 1879: 196.

*Eucynortaquadripustulata* — Roewer 1912: 55. — Ahumada-C. et al. 2022: 2, figs 1–4, 5D, 6.

**Distribution.** Atlántico, Bolívar, Cesar, La Guajira, Magdalena. Amazon-Orinoco-Southern Caribbean mangroves (NT1401), Guajira-Barranquilla xeric scrub (NT1308), Magdalena-Urabá moist forests (NT0137), Cordillera Oriental montane forests (NT0118) and Sinú Valley dry forests (NT0229).

***Eucynortavenosa* Roewer, 1928**

**Distribution.** Chocó. Chocó-Darién moist forests (NT0115).

***Eucynortellaorbicularis* Roewer, 1947**

**Distribution.** Unknown department.

**Remarks.**Flórez and Sánchez-C (1995) refers to the Boyacá or Santander departments as possible localities.

***Eucynortulaypsilon* Roewer, 1925**

**Distribution.** Chocó. Chocó-Darién moist forests (NT0115).

***Eulibitiacastor* Medrano & Kury, 2017**

*Eulibitiacastor* Medrano & Kury, 2017: 8, figs 5–7, 34.

**Distribution.** Boyacá. Cordillera Oriental montane forests (NT0118).

***Eulibitiachacuamarei* Pinzón, Damron & Pinto-da-Rocha, 2021**

*Eulibitiachacuamarei*Pinzón et al., 2021: 205, figs 1, 2.

**Distribution.** Casanare. Llanos (NT0709).

***Eulibitiaclytemnestra* Medrano & Kury, 2017**

*Eulibitiaclytemnestra* Medrano & Kury, 2017: 12, figs 8–10, 33.

**Distribution.** Santander. Magdalena Valley montane forests (NT0136).

***Eulibitiaectroxantha* (Mello-Leitão, 1941)**

*Brachylibitiaectroxantha* Mello-Leitão, 1941: 166, fig. 1.

*Cynortaectroxantha* — Goodnight and Goodnight 1953: 38.

*Platymessaectroxantha* — Medrano and Kury 2016: 57. — Tagged as species inquirenda.

*Eulibitiaectroxantha* — Medrano and Kury 2017: 16, fig. 54. — Medrano et al. 2019: 346, figs 1–4.

**Distribution.** Boyacá. Northern Andean páramo (NT1006) and Magdalena Valley montane forests (NT0136).

***Eulibitiahelena* Medrano & Kury, 2017**

*Eulibitiahelena* Medrano & Kury, 2017: 17, figs 11–14, 34.

**Distribution.** Norte de Santander. Cordillera Oriental montane forests (NT0118).

***Eulibitiah-inscriptum* (Mello-Leitão, 1941)**

*Platymessah-inscripta* Mello-Leitão, 1941: 167, fig. 2.

*Platymessanigrolimbata* Mello-Leitão, 1941: 168, fig. 3. — B. Soares 1945: 344.

*Cynortah-inscripta* — H. Soares 1970: 325.

*Platymessah-inscriptum* — Kury and Alonso-Zarazaga 2011: 51. — Medrano and Kury 2016: 58, figs 1–29.

*Eulibitiah-inscriptum* — Medrano and Kury 2017: 17, fig. 32.

**Distribution.** Boyacá and Santander. Magdalena Valley montane forests (NT0136) and Northern Andean páramo (NT1006).

***Eulibitialeda* Medrano & Kury, 2017**

*Eulibitialeda* Medrano & Kury, 2017: 22, figs 15–18, 33.

**Distribution.** Santander. Magdalena Valley montane forests (NT0136).

***Eulibitiamaculata* Roewer, 1912**

*Eulibitiamaculata* Roewer, 1912: 17, pl. 1, fig. 1. — Roewer 1923: 298, fig. 320. — Sørensen in Henriksen 1932: 389. — B. Soares 1945: 343. — Weidner 1959: 122. — Pinto-da-Rocha and Hara 2011: 2. — Medrano and Kury 2017: 27, figs 3, 19–22, 32.

Libitia (Messa) castanea Sørensen in Henriksen, 1932: 415.

*Paramessacastanea* — Mello-Leitão 1933: 109.

*Messatanacastanea* — Strand 1942: 398.

**Distribution.** Boyacá, Cundinamarca, and Tolima. Northern Andean páramo (NT1006) and Magdalena Valley montane forests (NT0136).

***Eulibitiapollux* Medrano & Kury, 2017**

*Eulibitiapollux* Medrano & Kury, 2017: 34, figs 23–26, 31B, 34.

**Distribution.** Boyacá. Magdalena Valley montane forests (NT0136).

***Eulibitiascalaris* (Sørensen, 1932)**

Libitia (Messa) scalaris Sørensen in Henriksen, 1932: 414.

*Acromareslateralis* Goodnight & Goodnight, 1943: 2, fig. 7. — Flórez and Sánchez-C 1995: 368.

*Messascalaris* — Mello-Leitão 1933: 112.

*Messatanascalaris* — Strand 1942: 398.

*Eulibitiascalaris* — Kury and Medrano 2017: 40, figs 27–30, 31A, 33.

*Cynortalateralis* — Goodnight and Goodnight 1953: 38.

**Distribution.** Cundinamarca. Magdalena Valley montane forests (NT0136).

***Eulibitiavictoriae* (Pinzón-M. & Townsend, 2017)**

*Platymessavictoriae*Pinzón-M. et al., 2017: 61, figs 1–7.

*Eulibitiavictoriae* — Kury et al. 2020: 14.

**Distribution.** Cesar. Cordillera Oriental montane forests (NT0118).

***Flirteaalpha* (Sørensen, 1932)**

**Distribution.** Cundinamarca. Magdalena Valley montane forests (NT0136).

***Flirteafusca* (Sørensen, 1932)**

**Distribution.** Unknown department.

***Flirteagranulosa* (Simon, 1879)**

**Distribution.** Cundinamarca. Magdalena Valley montane forests (NT0136).

***Flirtealimbata* (Sørensen, 1932)**

**Distribution.** Unknown department.

***Flirteamilitaris* (Simon, 1879)**

**Distribution.** Cundinamarca. Magdalena Valley montane forests (NT0136).

***Flirteatuberculata* (Sørensen, 1932)**

**Distribution.** Unknown department.

***Flirteaventricosa* (Simon, 1879)**

**Distribution.** Cundinamarca. Magdalena Valley montane forests (NT0136).

***Libitiabipunctata* Sørensen, 1932**

Libitia (Libitia) bipunctata Sørensen in Henriksen, 1932: 417.

*Libitiabipunctata* — Mello-Leitão 1933: 109. — Medrano, et al. 2020: 5, figs 1–4, 14.

*Libitiellabipunctata* — Roewer 1947: 8, pl. 1, fig. 3.

**Distribution.** Cundinamarca. Magdalena Valley montane forests (NT0136), Cordillera Oriental montane forests (NT0118) and Northern Andean páramo (NT1006).

***Libitiacordata* (Gervais, 1844)**

*Cosmetuscordatus* Gervais, 1844: 117, pl. 46, fig. 9.

*Libitiacordata* — Butler 1873: 115. — Simon 1879: 216. — Roewer 1912: 12; 1923: 293. — Mello-Leitão 1923: 108; 1932: 53; 1933: 109. — Medrano et al. 2020: 10, figs 5–8, 14.

Libitia (Libitia) cordata — Sørensen in Henriksen 1932: 419.

**Distribution.** Cundinamarca. Magdalena Valley montane forests (NT0136) and Northern Andean páramo (NT1006).

***Libitiagandalf* Medrano, Ázara & Kury, 2020**

*Libitiagandalf*Medrano et al., 2020: 14, figs 9, 10, 14.

**Distribution.** Meta. Cordillera Oriental montane forests (NT0118).

***Libitiaiguaque* Medrano, Ázara & Kury, 2020**

*Libitiaiguaque*Medrano et al., 2020: 16, figs 11, 12, 14.

**Distribution.** Boyacá. Northern Andean páramo (NT1006).

***Metacynortabella* Roewer, 1963**

**Distribution.** Putumayo. Napo moist forests (NT0142).

***Meterginusaffinis* Roewer, 1963**

**Distribution.** Putumayo. Napo moist forests (NT0142).

***Meterginusflavicinctus* (Gervais, 1842)**

**Distribution.** Cundinamarca. Magdalena Valley dry forests (NT0221) and Magdalena Valley montane forests (NT0136).

***Meterginusmarginellus* (Simon, 1879)**

**Distribution.** Unknown department.

***Meterginusmarmoratus* (Roewer, 1912)**

**Distribution.** Cundinamarca. Magdalena Valley montane forests (NT0136).

***Meterginusobscurus* (Sørensen, 1932)**

**Distribution.** Unknown department.

***Meterginusprosopis* Roewer, 1912**

**Distribution.** Cundinamarca, Meta, and Tolima. Magdalena Valley montane forests (NT0136) and Cordillera Oriental montane forests (NT0118).

***Meterginussimonis* (With, 1932)**

Rhaucus (Erginus) simonis With in Henriksen, 1932: 350.

*Meterginussimonis* — Mello-Leitão 1933: 110. — Kury 2003: 73. — Kury et al. 2020: 17.

**Distribution.** Unknown department.

***Meterginustogatus* (Sørensen, 1932)**

**Distribution.** Cundinamarca. Magdalena Valley montane forests (NT0136).

***Paecilaemaaltaspinulatum* Goodnight & Goodnight, 1943**

**Distribution.** Unknown department.

***Paecilaemaatroluteum* Roewer, 1912**

**Distribution.** Atlántico. Guajira-Barranquilla xeric scrub (NT1308).

***Paecilaemacontextum* Roewer, 1928**

**Distribution.** Unknown department.

***Paecilaemadistinctum* Roewer, 1915**

**Distribution.** Atlántico. Guajira-Barranquilla xeric scrub (NT1308).

***Paraprotusatroluteus* Roewer, 1912**

**Distribution.** Atlántico. Guajira-Barranquilla xeric scrub (NT1308).

***Protusspeciosus* (Roewer, 1927)**

*Paraprotusspeciosus* Roewer, 1927: 628, pl. 1, fig. 11.

*Protusspeciosus* — Medrano et al. 2021a: 52.

**Distribution.** Amazonas. Solimões-Japurá moist forests (NT0163).

**Remarks.** The type locality for this species is Maranhão state, in Brazil. It was recorded for Colombia by Medrano et al. (2021a).

***Rhaucoidesnasa* Medrano, García & Kury, 2022**

*Rhaucoidesnasa*Medrano et al., 2022: 196, figs 7–9, 22.

**Distribution.** Cauca. Magdalena Valley montane forests (NT0136).

***Rhaucoidesriveti* Roewer, 1914**

*Rhaucoidesriveti* Roewer, 1914: 125, pl. 13, fig. 3. — Roewer 1923: 306, fig. 333.

*Rhaucoidesfestae* Roewer, 1925: 3, pl. 5, fig. 1. — *Pararhauculussulfureus* Mello-Leitão, 1939: 171. — Established as a junior subjective synonym of *Rhaucoidesfestae* Roewer, 1925 by Kury (2003).

*Cumbaliaoctomaculata* Roewer, 1963: 51, pl. 9, fig. 13.

**Distribution.** Nariño. Northwestern Andean montane forests (NT0145).

***Rhaucusflorezi* García & Kury, 2017**

*Rhaucusflorezi* García & Kury, 2017: 426, figs 1e, 15–17, 18e, 23.

**Distribution.** Boyacá. Magdalena Valley montane forests (NT0136).

***Rhaucuspapilionaceus* (Simon, 1879)**

*Erginuspapilionaceus* Simon, 1879: 205.

*Flirteapapilionacea* — Roewer 1912: 77. — Roewer 1923: 347. — Roewer 1927: 593. — Mello-Leitão 1932: 78.

Rhaucus (Erginus) papilionaceus — Henriksen 1932: 352.

*Rhaucuspapilionaceus* — García and Ahumada-C. 2018: 201, figs 1–8.

**Distribution.** Santander. Magdalena Valley montane forests (NT0136).

***Rhaucusquinquelineatus* Simon, 1879**

*Rhaucusquinquelineatus* Simon, 1879: 215. — Sørensen in Henriksen 1932: 358. — García and Kury 2017: 413, figs 1d, 6–8, 20a, 22.

*Pararhaucusobscurus* Pickard-Cambridge, 1905: 572. — Roewer 1912: 102. — Roewer 1923: 378.

*Metarhaucusalbilineatus* Roewer, 1912: 147, pl. 7, figs 5, 6. — Flórez and Sánchez-C 1995: 368.

Rhaucus (Rhaucus) muticus Sørensen, 1932: 360.

Rhaucus (Rhaucus) tristis — Sørensen 1932: 363, fig. 26.

*Flirteaquinquelineata* — Roewer 1912: 76. — Roewer 1923: 346, fig. 393. — Mello-Leitão 1923: 112. — Roewer 1927: 594. — Mello-Leitão 1932: 79, fig. 47. — B. Soares 1945: 343. — Roewer 1963: 58. — Flórez and Sánchez-C 1995: 368.

*Paecilaemaobscurum* — Goodnight and Goodnight 1953: 54. — Kury 2003: 78.

*Flirteamutica* — Mello-Leitão 1933: 110.

*Flirteatristis* — Mello-Leitão 1933: 110.

**Distribution.** Boyacá and Cundinamarca. Northern Andean páramo (NT1006), Magdalena Valley montane forests (NT0136) and Cordillera Oriental montane forests (NT0118).

***Rhaucusrobustus* (Mello-Leitão, 1941)**

*Megarhaucusrobustus* Mello-Leitão, 1941: 169, fig. 4.

*Rhaucusrobustus* — García & Kury, 2017: 422, figs 1c, 12–14, 18d, 23.

**Distribution.** Boyacá and Santander. Northern Andean páramo (NT1006) and Magdalena Valley montane forests (NT0136).

***Rhaucusserripes* (Simon, 1879)**

*Erginusserripes* Simon, 1879: 204.

*Metarhaucusfuscus* Pickard-Cambridge, 1905: 572.

*Metarhaucusreticulatus* Roewer, 1912: 145, pl. 7, fig. 4.

Rhaucus (Rhaucus) geographicus Sørensen, 1932: 369.

*Flirteapaucimaculata* Roewer, 1963: 58, fig. 24. — Flórez and Sánchez-C 1995.

*Flirteaserripes* — Roewer 1912: 77; 1923: 348, figs 396–397; 1927: 593. — Mello-Leitão 1932: 78.

Rhaucus (Erginus) serripes — Henriksen in Sørensen 1932: 352.

*Rhaucusserripes* — García and Kury 2017: 417, figs 1f, 9–11, 18c, 23.

*Erginusfuscus* — Roewer, 1912: 68.

*Metarhaucusfuscus* — Roewer 1923: 342; 1927: 588.

*Erginusreticulatus* — Roewer 1912: 68.

*Metarhaucusreticulatus* — Roewer 1923: 343, fig. 387; 1927: 588; 1959: 80. — Forcart 1961: 51. — Flórez and Sánchez-C 1995: 368.

*Flirteageographica* — Mello-Leitão 1933: 110.

**Distribution.** Boyacá and Cundinamarca. Northwestern Andean montane forests (NT0145), Magdalena Valley montane forests (NT0136), Cordillera Oriental montane forests (NT0118) and Northern Andean páramo (NT1006).

***Rhaucusvulneratus* Simon, 1879**

*Rhaucusvulneratus* Simon, 1879: 213. Pickard-Cambridge 1905: 572. — Roewer 1912: 78. — Roewer 1923: 349, fig. 400. — Mello-Leitão 1923: 113. — Mello-Leitão 1932: 57, fig. 23. — Mello-Leitão 1933: 110. — García and Kury 2017: 405, figs 1a, b, 2–5, 18a, 19a, b, 22.

*Neorhaucusaurolineatus* Pickard-Cambridge, 1905: 572.

Rhaucus (Rhaucus) vulneratus — Sørensen 1932: 355.

*Raucusvulneratus* — González-Sponga 1992: 427.

*Neorhaucusaurolineatus* — Roewer 1912: 25. — Roewer 1923: 305.

**Distribution.** Boyacá and Cundinamarca. Magdalena Valley montane forests (NT0136) and Northern Andean páramo (NT1006).

***Sibambeacincta* (Perty, 1833)**

*Discosomacincta* Perty, 1833: 209, pl. 40, fig. 6.

*Discosomaticussturmi* Roewer, 1963: 58, fig. 25.

*Sibambeacincta* — Medrano et al. 2021a: 56.

**Distribution.** Amazonas and Vaupés. Purus várzea (NT0156), Iquitos várzea (NT0128), Solimões-Japurá moist forests (NT0163) and Caqueta moist forests (NT0107).

**Remarks.** The type locality for this species is the state of Bahia, in Brazil. It was recorded for Colombia by Medrano et al. (2021a).

***Taitoinsperatus* (Soares, 1970)**

*Cynortainsperata* Soares, 1970: 323, fig. 3. — Kury 2003: 45.

*Taitoinsperatus* — Kury and Barros 2014: 36, figs 1d, 6ae–ai, 17d–f, 28.

**Distribution.** Amazonas. Purus várzea (NT0156).

***Taitomedinae* Kury & Barros, 2014**

*Taitomedinae* Kury & Barros, 2014: 39, figs 1a, 17j–l, 26a–g, 35.

**Distribution.** Amazonas. Solimões-Japurá moist forests (NT0163).

***Taitooblongatus* (Roewer, 1928)**

*Cynortulaoblongata* Roewer, 1928: 576, fig. 28. — Mello-Leitão 1932: 58. — Kury 2003: 52.

*Taitooblongatus* — Kury and Barros 2014: 40, figs 1e, 6a–l, 17a–c, 29.

**Distribution.** Meta. Cordillera Oriental montane forests (NT0118).

***Zaraxoliadevians* (Sørensen, 1932)**

**Distribution.** Unknown department.

##### Cranaidae Roewer, 1913

***Allocranauscolumbianus* Roewer, 1915**

**Distribution.** Quindío. Magdalena Valley montane forests (NT0136).

***Cranausalbipustulatus* Roewer, 1943**

**Distribution.** La Guajira. Guajira-Barranquilla xeric scrub (NT1308).

***Cranauschlorogaster* (Gervais, 1844)**

**Distribution.** Unknown department.

***Cranauscinnamomeus* (Gervais, 1844)**

**Distribution.** Unknown department.

***Deriacrussimoni* Roewer, 1932**

**Distribution.** Cundinamarca. Magdalena Valley montane forests (NT0136).

***Holocranauscalcar* (Roewer, 1912)**

**Distribution.** Antioquia. Cauca Valley montane forests (NT0109) and Magdalena Valley montane forests (NT0136).

***Holocranauscalus* (Goodnight & Goodnight, 1944)**

**Distribution.** Valle del Cauca. Cauca Valley montane forests (NT0109).

***Holocranauslongipes* Roewer, 1913**

*Holocranauslongipes* Roewer, 1913: 400, fig. 158. — Roewer 1923: 556, fig. 695. — 1932: 293, fig. 9. — Soares and Soares 1948: 602.

*Phareicranausgiganteus* Roewer, 1932: 299. — Kury 2003: 93. — Pinto-da-Rocha and Bonaldo 2011: 27.

**Distribution.** Unknown department.

**Remarks.** The type locality for this species is the Aguacatal River, Colombia. The locality is not precise (Kury 2003).

***Holocranauspectinitibialis* (Roewer, 1914)**

*Tolimaiuspectinitibialis* Roewer, 1914: 125, figs 12–13. — Roewer 1923: 558, fig. 698.

*Holocranauspectinitibialis* — Soares and Soares 1948: 603. — Kury 2003: 93.

**Distribution.** Tolima. Magdalena Valley montane forests (NT0136).

***Holocranaussimplex* Roewer, 1913**

**Distribution.** Unknown department.

***Homocranaustetracalcar* Roewer, 1915**

**Distribution.** Quindío. Magdalena Valley montane forests (NT0136).

***Isocranausgorgonae* Hirst, 1926**

**Distribution.** Nariño. Chocó-Darién moist forests (NT0115).

***Isocranausobscurus* Roewer, 1915**

**Distribution.** Meta. Apure-Villavicencio dry forests (NT0201).

***Megacranausareolatus* Roewer, 1932**

**Distribution.** Tolima.

***Megacranauspygoplus* Roewer, 1913**

**Distribution.** Antioquia. Northern Andean páramo (NT1006).

***Metacranaustricalcaris* Roewer, 1913**

**Distribution.** Antioquia. Cauca Valley montane forests (NT0109).

***Neocranausalbiconspersus* Roewer, 1913**

**Distribution.** Maracaibo (without further specific locality data).

**Remark.** Several localities are called Maracaibo in Colombia and Venezuela. Kury (2003) suggests that it could be a Venezuelan locality. The other species in the genus is from Ecuador, and a taxonomic revision is underway to better understand the distribution and composition of the genus. At the moment, it is impossible to associate the species with any of the localities.

***Neocranausarmatissimus* (Mello-Leitão, 1941)**

*Mitobatulinaarmatissima* Mello-Leitão, 1941: 170, fig. 5. — Soares and Soares 1949: 235.

*Neocranausarmatissimus* — Kury 2003: 95.

**Distribution.** Nariño, Napo moist forests (NT0142).

***Panalusrobustus* Goodnight & Goodnight, 1947**

**Distribution.** Cauca. Cauca Valley montane forests (NT0109).

***Paracranauscrassipalpis* Roewer, 1913**

**Distribution.** Atlántico. Guajira-Barranquilla xeric scrub (NT1308).

***Peripasimplex* Roewer, 1932**

**Distribution.** Cundinamarca. Magdalena Valley montane forests (NT0136).

***Phalangodusanacosmetus* Gervais, 1842**

*Phalangodusanacosmetus* Gervais, 1842: 3, pl. 4; 1844: 114, pl. 46, fig. 3. — Erichson 1845: 267. — Thorell 1877: 115. — Simon 1879: 241. — Roewer 1913: 138, fig. 63. — 1923: 446, fig. 562. — Kästner 1937: 300, fig. 364. — Soares and Soares 1954: 289. — Kury 1996: 178, figs 1–4. — Acosta 1996: 224. — Hara et al. 2014: 569. — Villarreal and García 2016: 5, figs 1–3, 16a, 18–19.

*Allocranausgiganteus* Mello-Leitão, 1940: 307, fig. 8. —.B. Soares 1945: 349. — Soares and Soares 1948: 587.

**Distribution.** Cundinamarca. Magdalena Valley montane forests (NT0136).

***Phalangodusbriareos* Villarreal & García, 2016**

*Phalangodusbriareos* Villarreal & García, 2016: 9, figs 4–6, 7a–d, 18, 19. — Barriga et al. 2019: 117.

**Distribution.** Santander. Magdalena Valley montane forests (NT0136).

***Phalangodusandresi* sp. nov.**

**Distribution.** Cundinamarca. Chocó-Darién Moist Forests (NT0115).

***Phalangoduscottus* Villarreal & García, 2016**

*Phalangoduscottus* Villarreal & García, 2016: 15, figs 8a, b, 9, 10, 18.

**Distribution.** Meta. Apure-Villavicencio dry forests (NT0201).

***Phalangodusgyes* Villarreal & García, 2016**

*Phalangodusgyes* Villarreal & García, 2016: 21, figs 7e–f, 8c–d, 11, 12, 18.

**Distribution.** Tolima. Magdalena Valley montane forests (NT0136).

***Phalangoduskuryi* Villarreal & García, 2016**

*Phalangoduskuryi* Villarreal & García, 2016: 25, figs 13–15, 18.

**Distribution.** Magdalena. Guajira-Barranquilla xeric scrub (NT 1308).

***Phareicranausalbigranulatus* Roewer, 1913**

*Phareicranausalbigranulatus* Roewer, 1913: 404, fig. 160. — Kury 2003: 96. — Pinto-da-Rocha and Bonaldo 2011: 27.

**Distribution.** Tolima. Magdalena Valley montane forests (NT0136).

**Remarks.**Pinto-da-Rocha and Bonaldo (2011) question the generic placement of this species.

***Phareicranausalbigyratus* Roewer, 1932**

*Phareicranausalbigyratus* Roewer, 1932: 303. — Kury 2003: 96. — Pinto-da-Rocha and Bonaldo 2011: 6.

**Distribution.** Cundinamarca. Magdalena Valley montane forests (NT0136).

***Phareicranausangelicus* (Roewer, 1963)**

*Santineziaangelica* Roewer, 1963: 69. — Pinto-da-Rocha and Kury 2003: 182. — Kury 2003: 97.

*Phareicranausangelicus* — Pinto-da-Rocha and Bonaldo 2011: 8, fig. 4B, C.

**Distribution.** Meta. Cordillera Oriental montane forests (NT0118).

***Phareicranausspinulatus* (Goodnight & Goodnight, 1943)**

*Santineziaspinulata* Goodnight & Goodnight, 1943: 9, figs 26–28. — Pinto-da-Rocha and Kury 2003: 177, 197, 198, 202, 204, 205, fig. 51. — Kury 2003: 99.

*Phareicranausspinulatus* — Pinto-da-Rocha and Bonaldo 2011: 26, fig. 18C, D.

**Distribution.** Unknown department.

***Sibundoxiascripta* Roewer, 1963**

**Distribution.** Putumayo. Eastern Cordillera real montane forests (NT0121).

***Stygnicranausalessandroi* Orrico & Kury, 2009**

*Stygnicranausalessandroi* Orrico & Kury, 2009: 474, figs 2–5.

**Distribution.** Valle del Cauca. Cauca Valley montane forests (NT0109).

***Stygnicranausponcedeleoni* Orrico & Kury, 2009**

*Stygnicranausponcedeleoni* Orrico & Kury, 2009: 476, figs 6–11.

**Distribution.** Valle del Cauca. Northwestern Andean montane forests (NT0146).

***Tetracranauszilchi* Roewer, 1963**

**Distribution.** Valle del Cauca. Northwestern Andean montane forests (NT0146) and Magdalena Valley montane forests (NT0136).

***Ventrifurcaalbipustulata* Roewer, 1913**

*Ventrifurcaalbipustulata* Roewer, 1913: 383, figs 149–150. — Kury 2003: 100. — Villarreal et al. 2015: 6, figs 1, 5, 7, 8, 10, 11, 13.

*Microcranauspustulatus* Roewer, 1913: 353, fig. 137. — Kury 2003: 93.

*Cayabeusperlatus* Roewer, 1932: 337, fig. 53. — Kury 2003: 91.

**Distribution.** Antioquia. Cauca Valley montane forests (NT0109).

***Ventrifurcacaffeinica* Villarreal, Kury & Pinto-da-Rocha, 2015**

*Ventrifurcacaffeinica*Villarreal et al., 2015: 11, figs 2, 4, 6, 12, 13.

**Distribution.** Quindío. Cauca Valley montane forests (NT0109).

***Ventrifurcadybasi* (Goodnight & Goodnight, 1947)**

*Rhopalocranausdybasi* Goodnight & Goodnight, 1947: 40, fig. 20.

*Neocranausdybasi* — Kury 2003: 95.

*Ventrifurcadybasi* — Villarreal et al. 2015: 14, fig. 3A, B, C.

**Distribution.** Valle del Cauca. Cauca Valley montane forests (NT0109).

**Remarks.** Generic assignment from *Neocranaus* by Villarreal et al. (2015).

***Ventrifurcaphallaina* sp. nov.**

**Distribution.** Valle del Cauca. Chocó-Darién moist forests (NT0115).

***Ventrisudismira* Roewer, 1963**

**Distribution.** Huila. Patía Valley dry forests (NT0225).

##### Manaosbiidae Roewer, 1943

***Camelianusfuhrmanni* Roewer, 1912**

**Distribution.** Antioquia. Cauca Valley montane forests (NT0109) and Magdalena Valley montane forests (NT0136).

***Cucutacolanigra* Mello-Leitão, 1940**

**Distribution.** Norte de Santander. Catatumbo moist forests (NT0108).

***Gonogotusareolatus* Roewer, 1943**

**Distribution.** Cundinamarca. Magdalena Valley montane forests (NT0136).

***Rhopalocranausapiculatus* Roewer, 1932**

**Distribution.** Unknown department.

***Rhopalocranausatroluteus* Roewer, 1913**

**Distribution.** Tolima. Magdalena Valley montane forests (NT0136).

***Rhopalocranauscolumbianus* (Roewer, 1963)**

*Microcranauscolumbianus* Roewer, 1963: 64, figs 34, 35.

*Rhoplocranauscolumbianus* — Villarreal et al. 2015: 14, fig. 15.

**Distribution.** Cauca. Patía Valley dry forests (NT0225).

***Rhopalocranausypsilon* Roewer, 1913**

**Distribution.** Tolima. Magdalena Valley montane forests (NT0136).

***Semostrustarsalis* Roewer, 1943**

**Distribution.** Cundinamarca. Magdalena Valley montane forests (NT0136).

##### Nomoclastidae Roewer, 1943

***Callcosmabarasana* Pinto-da-Rocha & Bragagnolo, 2017**

*Callcosmabarasana* Pinto-da-Rocha & Bragagnolo, 2017: 103, figs 4B, 8B, 10B, 13A, B.

**Distribution.**Vaupés. Caqueta moist forests (NT0107).

***Callcosmagracillima* Roewer, 1932**

*Callcosmagracillima* Roewer, 1932: 331, fig. 47. — Soares and Soares 1948: 591. — Kury 2003: 91. — Pinto-da-Rocha and Bragagnolo 2017: 110, figs 1E, 4D, 8D, 10C, 13E, F.

**Distribution.** Amazonas. Solimões-Japurá moist forests (NT0163).

***Nomoclastesquasimodo* Pinto-da-Rocha, 1997**

*Nomoclastesquasimodo* Pinto-da-Rocha, 1997: 171, figs 1–6, 449, 450, 587, 592, 596. – Kury 2003: 228.

**Distribution.** Cundinamarca. Magdalena Valley montane forests (NT0136).

***Nomoclastestaedifer* Sørensen, 1932**

*Nomoclastestaedifer* Sørensen, 1932: 300, fig. 11. — Roewer 1943: 36. — Pinto-da-Rocha 1997: 171, figs 7, 8. — Kury 2003: 228.

**Distribution.** Department unknown.

***Paraphalangodussynacanthus* Roewer, 1915, new family assignment**

**Distribution.** Tolima (without a specific locality).

**Remark.** The type locality for this species refers to Páramo del Tolima, located in the department of Tolima, which comprises four páramo complexes, namely Las Hermosas, Chilí Barragán, Nevado del Huila-Moras, and Los Nevados. Therefore, the specific location within the Tolima department cannot be determined without further information. Originally described as Gonyleptidae, Kury (2003) placed it as incertae sedis within Gonyleptoidea. Based on our study of a photograph of the holotype, we propose re-classifying it under Nomoclastidae, given the pedipalp morphology, absence of ornamentation in area I, and the shape of the dorsal shield type Zeta.

***Quindinabella* Roewer, 1915**

*Quindinabella* Roewer, 1915: 128, fig. 14. — Roewer 1923: 564, fig. 707. — Soares and Soares 1948: 615. — Flórez and Sánchez-C 1995: 369. — Kury 2003: 97. — Pinto-da-Rocha and Bragagnolo 2017: 114, figs 5D, 8H, 14A, B.

**Distribution.** Tolima. Magdalena Valley montane forests (NT0136).

***Quindinadiscolor* Pinzón & Pinto-da-Rocha, 2020**

*Quindinadiscolor* Pinzón & Pinto-da-Rocha, 2020: 542, figs 3, 4.

**Distribution.** Magdalena. Santa Marta montane forests (NT0159).

***Quindinahermesi* Pinzón & Pinto-da-Rocha, 2020**

*Quindinahermesi* Pinzón & Pinto-da-Rocha, 2020: 537, figs 1, 2.

**Distribution.** Atlántico, Bolívar. Magdalena-Urabá moist forests (NT0137) and Guajira-Barranquilla xeric scrub (NT 1308).

***Quindinamarginata* (Roewer, 1963)**

*Deriacrusmarginatus* Roewer, 1963: 68, fig. 42. — Flórez and Sánchez-C 1995: 368.

*Quindinamarginata* — Pinto-da-Rocha and Bragagnolo 2017: 120, figs 7A, 9E, 14G, H.

**Distribution.** Huila. Magdalena Valley montane forests (NT0136).

##### Stygnidae Simon, 1879

***Eutimesiusephippiatus* (Roewer, 1915)**

**Distribution.** Quindío. Magdalena Valley montane forests (NT0136).

***Eutimesiusornatus* (Roewer, 1943)**

**Distribution.** Cundinamarca. Magdalena Valley montane forests (NT0136).

***Eutimesiussimoni* Roewer, 1913**

**Distribution.** Putumayo. Napo moist forests (NT0142).

***Fortiajedi* Villarreal, Kury & Colmenares, 2022**

*Fortiajedi*Villarreal et al., 2022: 5, figs 1–11, 25–27.

**Distribution.** Magdalena. Santa Marta montane forests (NT0159).

***Fortiasith* Villarreal, Kury & Colmenares, 2022**

*Fortiasith*Villarreal et al., 2022: 8, figs 12–24, 28–30.

**Distribution.** Magdalena. Santa Marta montane forests (NT0159).

***Jabbastygnushuttorum* Kury & Villarreal, 2015**

*Jabbastygnushuttorum* Kury & Villarreal, 2015: 32, figs 4D–F, 17–19.

**Distribution.** Boyacá. Magdalena Valley montane forests (NT0136).

***Metaphareusalbimanus* Roewer, 1912**

**Distribution.** Tolima. Magdalena Valley montane forests (NT0136).

***Niceforoielusassimilis* Mello-Leitão, 1941**

**Distribution.** Norte de Santander. Catatumbo moist forests (NT0108).

***Obidosuscarnaval* (Villarreal-Manzanilla & Pinto-da-Rocha, 2006)**

*Protimesiuscarnaval* Villarreal-Manzanilla & Pinto-da-Rocha, 2006: 229, figs. 29–35, 44, 45.

*Obidosuscarnaval* — Villarreal et al. 2019: 229, figs 29–35, 44, 45.

**Distribution.** Amazonas. Solimões-Japurá moist forests (NT0163).

**Remarks.** The type locality for this species is Moa River in the state of Acre, in Brazil. It was recorded for Colombia by Villarreal-M. and Pinto-da-Rocha (2006).

***Phareusantrophilus* Villarreal & Rodríguez, 2006**

*Phareusantrophilus* Villarreal & Rodríguez, 2006: 103, figs 1–9.

**Distribution.** Santander. Magdalena Valley montane forests (NT0136).

***Phareusraptator* (Gervais, 1844)**

*Goniosomaraptator* Gervais, 1844: 107 (vol. 3), pl. 47, figs 1, 1a.

*Liophareusmamillatus* Mello-Leitão, 1940: 309, fig. 10. — B. Soares 1945: 386.

*Colomphareusrugosus* Goodnight & Goodnight, 1943: 10, figs 32–35. — Flórez and Sánchez-C 1995: 368.

*Allophareusrobustus* Roewer, 1963: 60, figs 26–28. — Flórez and Sánchez-C 1995: 368.

*Phareusraptator* — Simon 1879: 219. — Roewer 1912: 149. — 1913: 155, figs 68, 69. — 1923: 457, figs 475, 476. — Flórez and Sánchez-C 1995: 369. — Pinto-da-Rocha 1997: 220, figs 160–167, 489, 490, 582, 599. — Villarreal and Rodríguez 2006: 104.

**Distribution.** Cundinamarca. Cordillera Oriental montane forests (NT0118), Magdalena Valley montane forests (NT0136) and Northern Andean páramo (NT1006).

***Stygnusgertschi* (Roewer, 1963)**

**Distribution.** Cauca. Patía Valley dry forests (NT0225).

***Stygnuspectinipes* (Roewer, 1943)**

**Distribution.** Putumayo. Napo moist forests (NT0142).

***Stygnussimplex* (Roewer, 1913)**

**Distribution.** Putumayo. Napo moist forests (NT0142).

***Timesiusvesicularis* (Gervais, 1844)**

**Distribution.** Department unknown.

##### Samooidea Sørensen, 1886

**Stygnommatidae Roewer, 1923**

***Stygnommafuhrmanni* Roewer, 1912**

**Distribution.** Antioquia. Cauca Valley montane forests (NT0109).

##### Zalmoxoidea Sørensen, 1886

**Incertae sedis**

*Heveliacrucis* Kury, García & Ahumada-C., 2023

*Heveliacrucis*Kury et al. 2023: 39, figs 1–4.

**Distribution.** Bolívar. Magdalena-Urabá moist forests (NT0137).

##### Fissiphalliidae Martens, 1988

***Fissiphalliusspinulatus* Martens, 1988**

**Distribution.** Cundinamarca. Northern Andean paramo (NT1006).

***Fissiphalliussturmi* Martens, 1988**

**Distribution.** Cundinamarca. Magdalena Valley montane forests (NT0136).

***Fissiphalliussympatricus* Martens, 1988**

**Distribution.** Cundinamarca. Magdalena Valley montane forests (NT0136).

##### Icaleptidae Kury & Pérez, 2002

***Icaleptesmalkini* Kury & Pérez, 2002**

**Distribution.** Cesar. Santa Marta montane forests (NT0159).

##### Kimulidae Pérez-González, Kury & Alonso-Zarazaga, 2007

***Usatamainfumatus* Kury, García & Medrano, 2019**

*Usatama* Kury, García, and Medrano 2019: 239, figs 1–4.

**Distribution.** Cundinamarca. Cordillera Oriental montane forests (NT0118).

##### Zalmoxidae Sørensen, 1886

***Minuidesoedipus* Roewer, 1963**

*Minuidesoedipus* Roewer, 1963: 48, figs 6–8; — Šilhavý 1978: 62. — Flórez and Sánchez-C 1995: 369.

**Distribution.** Huila. Magdalena Valley dry forests (NT0221).

**Remarks.***Minuidesoedipus* Roewer, 1963 treated as a Kimulidae in Kury´s catalogue (Kury 2003); however, Pérez-González and Kury (2007) transferred *Minuides* Sørensen, 1932 to the Zalmoxidae.

***Stygnoleptesanalis* Banks, 1913**

**Distribution.** Department unknown.

***Stygnoleptescrassus* (Sørensen, 1932)**

**Distribution.** Department unknown.

***Timoleoncrassipes* Sørensen, 1932**

**Distribution.** Department unknown.

#### ﻿Rationale for exclusions

Two species, *Cosmetusflavopictus* Simon, 1880 and *Stygnoplusforcipatus* have traditionally been associated with the list of Colombian species, which we do not consider in our list. *Cosmetusflavopictus* was described for a locality in Darien, extrapolated by Kury (2003) as Colombian territory. Here, we follow the interpretation given by Medrano et al. (2021b) and consider not citing it as part of the Colombian species. *Stygnoplusforcipatus* Koch, 1845 was originally described without any locality data other than “Columb.” (Koch 1845). Subsequently, it was recorded from Venezuela and its presence in the Colombian fauna was called into question (Villarreal-M. and Rodríguez 2004). Other species of the genus *Stygnoplus* are known from the Caribbean region, in the Venezuelan Cordillera de La Costa and the Lesser Antilles, with the exception of *S.longipalpus* (Goodnight & Goodnight, 1942), which occurs in the Guiana Shield. Recently, the only Amazonian species originally described in this genus by Pinto-da-Rocha and Tourinho (2012) were transferred to *Yapacana* Pinto-da-Rocha, 1997 (Villarreal et al. 2021b). We have decided not to include *S.forcipatus* in the list at this time due to lack of evidence of its presence in the country.

### ﻿Diversity

**Figure 13.**
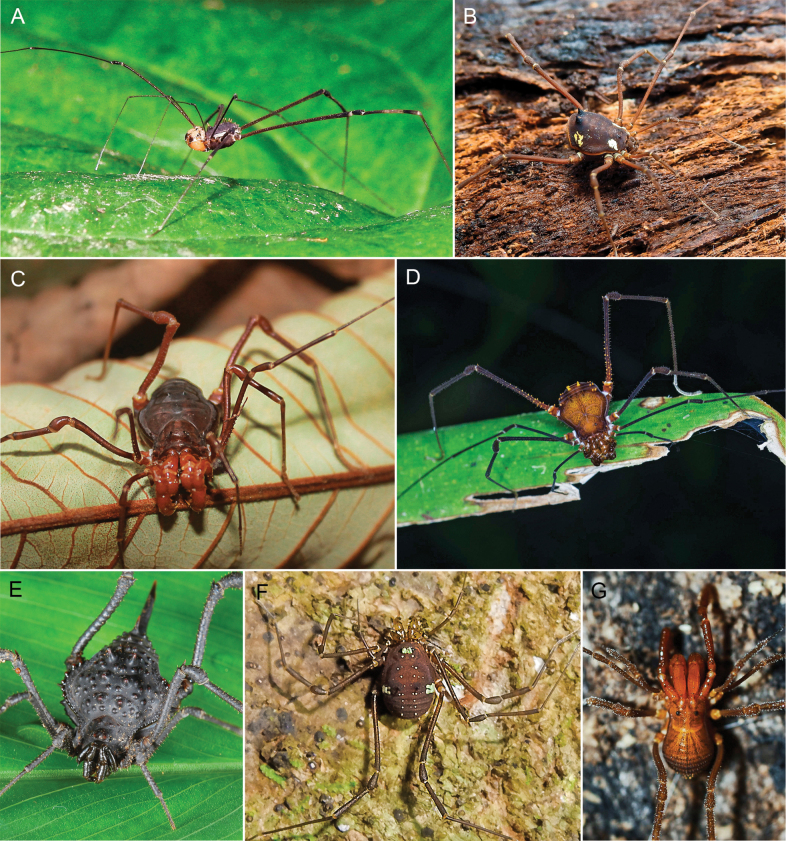
Selected Colombian harvestmen species, live specimens **A***Barinaspiragua* (Agoristenidae) from Bolívar **B***Eucynortaquadripustulata* (Cosmetidae) from Bolívar **C***Phalangoduscottus* (Cranaidae) from Cundinamarca **D***Phareicranaus* sp. (Cranaidae) from Chocó **E***Ampycustelifer* (Ampycidae) from Amazonas **F***Quindinahermesi* (Nomoclastidae) from Bolívar **G***Stygnomma* sp. (Stygnommatidae) from Tolima. Photographs by: Hugo Vides (**A, B, F**); Andrés García (**C**); Sarah Crews (**D**); Arthur Anker (**E**); Julio González / Entomopixel (**G**).

**Figure 14.**
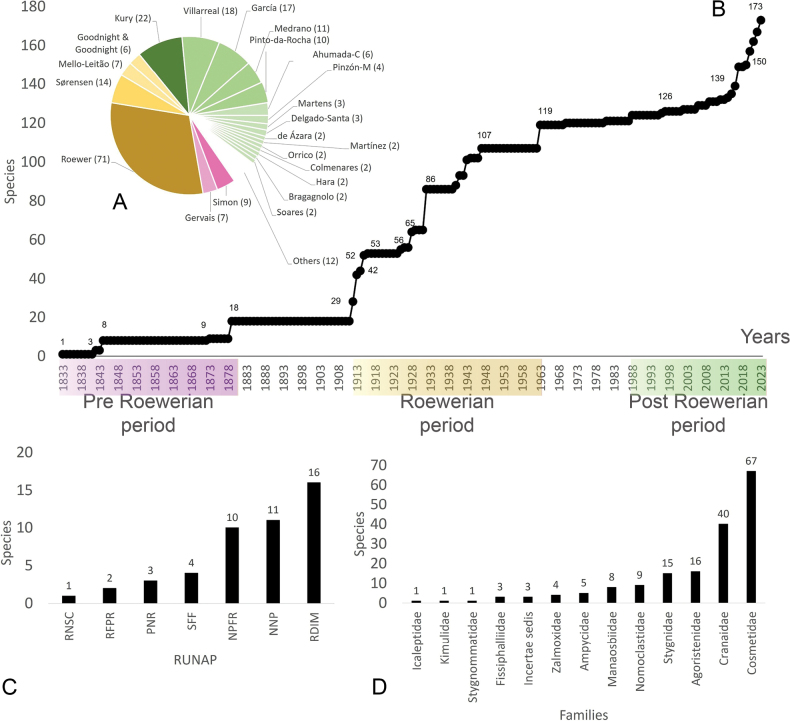
Laniatores species described from Colombia **A** the pie chart shows authors who described two or more species. For each author, every described species was counted, regardless of whether the authorship of the species is shared with other authors. For example, *Usatamainfumatus* was described by Kury, García & Medrano, 2019, and was counted as a species for each author. Authors with one species are included in the others category **B** accumulation curve of Laniatores species described from Colombia. Pink: descriptions made between 1833 and 1911; ochre: 1912–1963; green: 1964–2023 **C**Laniatores species in RUNAP **D** families of Laniatores species described from Colombia.

The Colombian opilionofauna of the suborder Laniatores is composed of 173 species, three of which are described in the present work. The 12 families listed in Colombia demonstrate the country’s high taxonomic diversity for this group. The most diverse family is Cosmetidae with 67 species (Fig. 13B), followed by Cranaidae with 40 species (Figs 13C, D, 14D). It is worth noting that a significant number of Laniatores species in Colombia are poorly documented, with only vague records for the country or department, and their precise type localities remain unknown. Specifically, there are 16 species of Cosmetidae, five species of Cranaidae, three species of Zalmoxidae, one species of Manaosbiidae, two species of Nomoclastidae, one species *Incertae sedis*, and one species of Stygnidae with uncertain recorded information. These findings highlight our limited understanding of the diversity and distribution of this group in Colombia.

Most records come from a few departments, indicating a sampling bias. Approximately 22% of the species are known from the department of Cundinamarca (38 spp.) near the capital of the country, followed by Tolima and Boyacá with 13 species each. In contrast, all six departments of the Amazon region have a combined total of only 15 recorded species. This exemplifies the limited knowledge we have on the diversity of harvestmen in the Colombian Amazon region. Of the 173 species of Laniatores known in Colombia, 35 are recorded without a specific locality; 28 of which have no information other than their presence in Colombia, and only seven have been located at a specific department (Table 2). The remaining departments have eight or fewer recorded species each, and eight departments have no records (Arauca, Caldas, Guainía, Guaviare, Risaralda, San Andrés y Providencia, Sucre, and Vichada).

**Table 2.** Number of Opiliones species by family recorded in WWF terrestrial ecoregions from Colombia. When the locality is unknown or not precise enough, the abbreviation DNA is used, and the record is not assigned to any ecoregion. DNA = does not apply.

WWF Ecoregion	Code (WWF)	Families	Species
Magdalena Valley montane forests	NT0136	Agoristenidae	6
		Cosmetidae	25
		Cranaidae	12
		Fissiphalliidae	2
		Manaosbiidae	5
		Nomoclastidae	4
		Stygnidae	6
Cordillera Oriental montane forests	NT0118	Agoristenidae	4
		Cosmetidae	10
		Cranaidae	1
		Kimulidae	1
		Stygnidae	1
Northern Andean páramo	NT1006	Cosmetidae	10
		Cranaidae	1
		Fissiphalliidae	1
		Stygnidae	1
Cauca Valley montane forests	NT0109	Cranaidae	9
		Manaosbiidae	1
		Stygnommatidae	1
Guajira-Barranquilla xeric scrub	NT1308	Agoristenidae	3
		Cosmetidae	4
		Cranaidae	3
		Nomoclastidae	1
Chocó-Darién moist forests	NT0115	Cosmetidae	7
		Cranaidae	2
		Ampycidae	1
Napo moist forests	NT0142	Agoristenidae	1
		Cosmetidae	2
		Cranaidae	1
		Stygnidae	3
Solimões-Japurá moist forests	NT0163	Cosmetidae	3
		Ampycidae	2
		Nomoclastidae	1
		Stygnidae	1
Santa Marta montane forests	NT0159	Agoristenidae	1
		Icaleptidae	1
		Nomoclastidae	1
		Stygnidae	2
Magdalena Valley dry forests	NT0221	Agoristenidae	1
		Cosmetidae	1
		Cranaidae	2
		Zalmoxidae	1
Northwestern Andean montane forests	NT0145	Cosmetidae	1
		Cranaidae	2
Apure-Villavicencio dry forests	NT0201	Cranaidae	2
		Ampycidae	1
Patía Valley dry forests	NT0225	Cranaidae	1
		Manaosbiidae	1
		Stygnidae	1
Catatumbo moist forests	NT0108	Agoristenidae	1
		Manaosbiidae	1
		Stygnidae	1
Magdalena-Urabá moist forests	NT0137	Agoristenidae	1
		Cosmetidae	1
		Incertae sedis	1
		Nomoclastidae	1
Sinú Valley dry forests	NT0229	Cosmetidae	2
Amazon-Orinoco-Southern Caribbean mangroves	NT1401	Agoristenidae	1
		Cosmetidae	1
Caqueta moist forests	NT0107	Cosmetidae	1
		Ampycidae	1
		Nomoclastidae	1
Purus várzea	NT0156	Cosmetidae	2
Eastern Cordillera real montane forests	NT0121	Cranaidae	1
Iquitos várzea	NT0128	Cosmetidae	1
Llanos	NT0709	Cosmetidae	1
Unknown Locality	DNA	Cosmetidae	21
		Cranaidae	6
		Manaosbiidae	1
		Nomoclastidae	1
		Stygnidae	1
		Zalmoxidae	3

**Figure 15.**
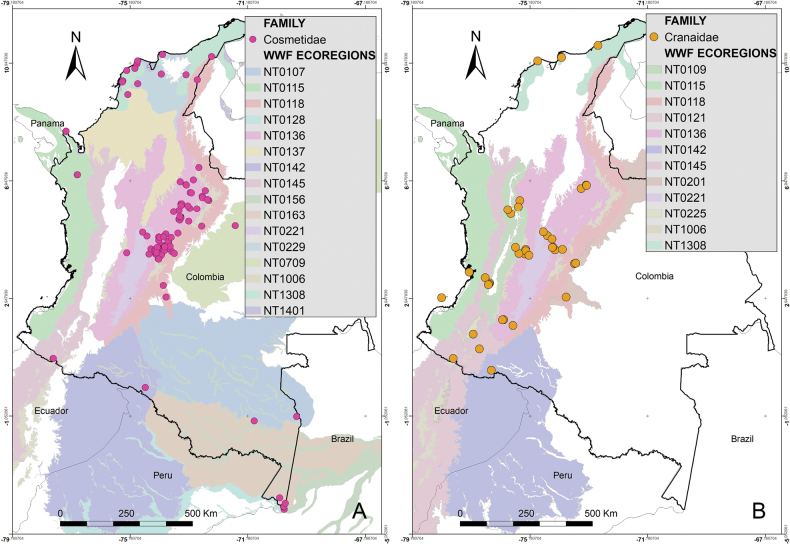
Geographical distribution of the families **A**Cosmetidae and **B**Cranaidae in Colombia. Colored polygons follow the regionalization of the Neotropical region proposed by WWF (Olson et al. 2001).

**Figure 16.**
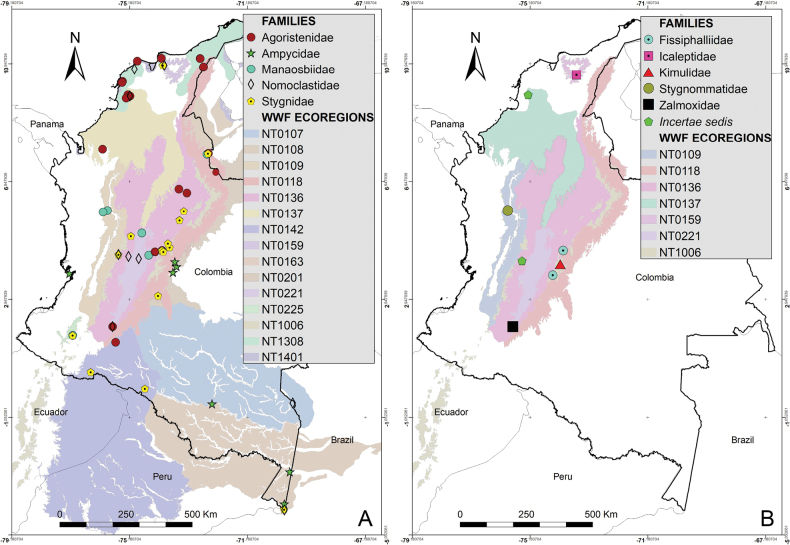
Geographical distribution of **A**Gonyleptoidea (Ampycidae, Manaosbiidae, Nomoclastidae and Stygnidae) and **B**Samooidea (Kimulidae, Stygnommatidae) and Zalmoxoidea (Icaleptidae, Fissiphalliidae, Zalmoxidae) in Colombia. Colored polygons follow the regionalization of the Neotropical region proposed by WWF (Olson et al. 2001).

Olson et al. (2001) recognized 35 ecoregions in Colombia, but only 22 of them have Laniatores species recorded (Table 2). The ecoregion with the highest number of species is the Magdalena Valley montane forests, with a total of 59 species. The ecoregions with the fewest species are Eastern Cordillera real montane forests, Iquitos várzea, Llanos, and Purus várzea, each with one species (Figs 15, 16). Only 21 species are found in more than one ecoregion, with *Eucynortaquadripustulata* (Fig. 13B) being the most widely recorded species, occurring in five ecoregions.

The Colombian NSPA lists 1,486 protected zones, of which 60 are NNP and FFS. The remaining areas are designated with some other category of protection (RUNAP 2022). Regarding OpilionesLaniatores, 36 species have been recorded from 29 areas, totaling 47 records, and 11 species have been recorded in seven NNP or FFS. The protection category with the highest number of the recorded species is RDIM, with 16 species, followed by NPFR with ten species, NNP with eight species, FFS with four species, PNR with three species, RFPR with two species, and RNSC with one species.

Similar to other neotropical countries, the taxonomic knowledge of harvestmen in Colombia is primarily based on the contributions of foreign authors. Remarkably Carl F. Roewer described almost half of the species known of the country. Therefore, for a pragmatic approach we divided the history into three periods: the first period prior to Roewer´s contributions, the second period in which most of the descriptions took place, led by Roewer’s works but with contributions from other authors, and finally, the third, post-Roewerian or modern period, with contributions from a diverse group of authors, including local authors for the first time. Our goal is not to present a detailed chronology, but rather to provide a general overview of the historical panorama and the individual contribution of the authors to the knowledge of the Colombian fauna. For a detailed chronicle and an in-depth look at the early history of opilionology, we recommend referring to Kury (2010), who presented an excellent compilation and analysis of papers between 1758 and 1804. Figure. 14B shows a timeline of descriptions of armored harvestmen species in Colombia and the contributions by author and periods.

Perty (1833) described *Sibambeacincta* (Perty, 1833), as the first armored harvestmen species known for Colombia. This is the starting point for the ancient or Pre-Roewerian period (1833–1880), during which 19 species were described by four authors, highlighting the contribution by French authors Eugène Simon (nine species described) and Paul Gervais (seven described species). The second period or so-called Roewerian period (1912–1963), is characterized by a substantial increase in the number of species described, thanks to the prolific work of the German author Roewer (71 Colombian species described between 1912 and 1963). Other important authors from this period include Mello-Leitão, Sørensen and Clarence J. and Marie L. Goodnight, totaling 27 described species. After Roewer’s death, there was a period of taxonomic inactivity for the Colombian fauna, until Martens (1988) published a paper on Colombian harvestmen, marking the beginning of the Modern or Post-Roewerian period (1988–present). Since then, contributions have become more frequent, particularly from Latin American authors, such as Brazilian authors A. B. Kury (22 spp.) and R. Pinto-da-Rocha (10 spp.) and Venezuelan author O. Villarreal (18 spp.), as well as local authors, highlighting A. F. García (17 spp.) and M. Medrano (11 spp.) (Fig. 14A).

## ﻿Discussion

### ﻿Diversity of Colombian Opiliones

Colombia is recognized as a megadiverse country, renowned for its numerous protected natural areas compared to other neotropical countries. Despite ongoing conservation efforts, there is a need to increase the knowledge on biodiversity in these areas to support their proper management and handling. Unhappily, the information available on Laniatores in these areas is still limited, despite the growing number of arachnologists in the region and the country, and the increasing research focus on local fauna (Fig. 14C).

The central and eastern cordillera are the most populated and accessible areas of Colombia. They are home to the largest universities and biological collections in the country and, consequently, the region with the highest number of descriptions and records of Laniatores (Figs 15, 16). Local researchers have made significant contributions to the study of Laniatores in the last decade, including the review of some genera such as *Rhaucus* or *Eulibitia* (García and Kury 2017; Medrano and Kury 2017) and description of some new species (e.g., Kury et al. 2019; García and Ahumada-C. 2022).

In contrast, the peripheral zones, like the Amazon, the Pacific, or the Caribbean, have received less attention from researchers. Nevertheless, recent efforts have been made to strengthen research in these regions. For instance, a school of arachnology at the Universidad del Valle, has emerged to study the Laniatores of Valle del Cauca in the Pacific region. Similarly, in the Caribbean, harvestmen have been the focus of some degree work, and recent collections have yielded specimens that have been added to the country’s biological collections, resulting in the descriptions of new species and new records (e.g., Pinzón and Pinto-da-Rocha 2020; Ahumada-C. et al. 2022; Kury et al. 2023).

**Figure 17.**
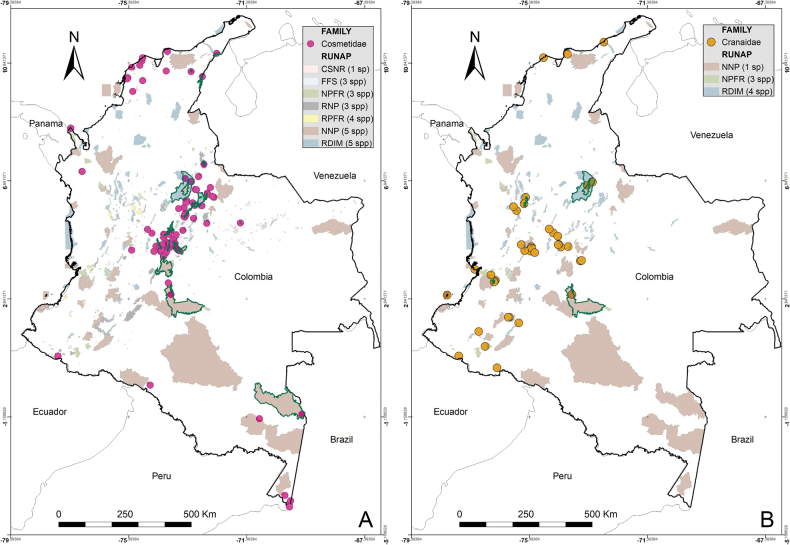
Geographical distribution of the families **A**Cosmetidae and **B**Cranaidae in Colombia. Colored polygons represent the RUNAP areas. Civil Society Nature Reserve (CSNR), Flora and Fauna Sanctuary (FFS), National Protective Forest Reserve (NPFR), Regional Natural Park (RNP), Regional Protective Forest Reserve (RPFR), National Natural Parks (NNP), Regional Integrated Management Districts (RDIM).

**Figure 18.**
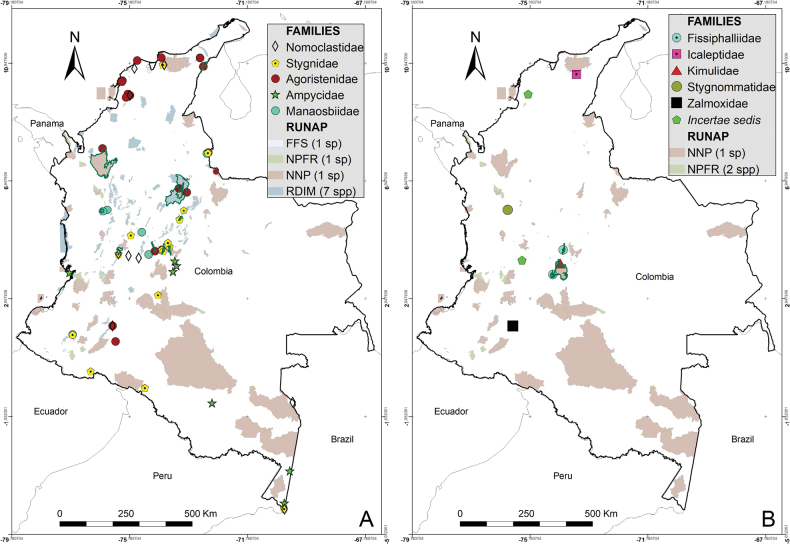
Geographical distribution of **A** the superfamilies Gonyleptoidea (Agoristenidae, Ampycidae, Manaosbiidae, Nomoclastidae and Stygnidae) and **B**Samooidea (Kimulidae, Stygnommatidae) and Zalmoxoidea (Icaleptidae, Fissiphalliidae, Zalmoxidae) in Colombia. Colored polygons represent the RUNAP areas. Flora and Fauna Sanctuary (FFS), National Protective Forest Reserve (NPFR), National Natural Parks (NNP), Regional Integrated Management Districts (RDIM).

Therefore, we believe that although the national inventory of Laniatores is far from being complete, further work on the local fauna will increase our knowledge of the group over time. Regarding the Colombian protected areas, the RNSC is the area with the lowest number of records of Laniatores (one species), while RDIM presents the highest number of records (16 species), probably because it is the only protected area in the country conceived as a rational use mode (Molina 2013), with research permits that are easier to access. However, when considering the total number of records for Laniatores across all categories of protected areas, studies are also scarce (Figs 17, 18). The implementation of strategies that facilitate the necessary regulatory procedures to collect specimens in protected natural areas, as well as increased investment in basic science projects, could help to generate a greater understanding of Colombian biodiversity in these areas. A successful case is the study by García and Medrano (2015) in the Reserva Natural Rio Ñambí, the only faunal study on harvestmen conducted in a protected natural area, resulting in the discovery of approximately 28 species new to science, demonstrating the hidden diversity in these areas, and the importance of inventories or taxonomic studies on the protected natural areas.

Continued exploration of ecosystems is imperative to search for Opiliones and achieve reliable taxonomic identifications, which are essential to determine the true distribution of the species. Such data is important in establishing their threat category, according whit the criteria from the International Union for Conservation of Nature (IUCN). This should be a goal for arachnology, given the high endemicity exhibited by these organisms and the significant anthropogenic pressures to which they are exposed.

### ﻿Taxonomic accounts

*Phalangodus* is an Andean cranaid genus whose species could be considered examples of short-range endemism, as defined by Harvey (2002). The distribution of *Phalangodusandresi* sp. nov. is within the area of generic distribution and represents the first case of sympatry in the genus, as it was collected in close proximity to *P.anacosmetus*, a widely distributed species found in the highlands of the department of Cundinamarca (Villarreal and García 2016).

*Phalangodusandresi* sp. nov. possesses all generic diagnostic characters presented by Villarreal and García (2016), and can be diagnosed by its smaller size, by having dorsal scutum tuberculated (shared only with *P.palpiconus*), being distinguished by the type of ornamentation of the femur IV of the males (refer to the diagnosis for further details). Unfortunately, of the type locality for *P.palpiconus* was erroneously attributed to Chile, which is unlikely due to the distribution pattern of the genus and even the family, as discussed by Hara et al. (2014), making impossible to establish any biogeographic relationship between both species.

Moreover, the family Ampycidae is known to have a little-explored diversity, primarily in Ecuador and Colombia. However, the taxonomic assignment of new species is a challenge for taxonomists due to the paucity of old descriptions and poor generic diagnosis. In addition, many species exhibit similar external morphology, and for more species, genitalia remain unknown, with just some exceptions (e.g., Villarreal and Kury 2012; Kury and Quintero 2014; Tourinho and Mendes 2014; Hara et al. 2017). Despite these challenges, recent studies on some species have shed light on the external morphology and genitalia of the subfamily. Further research is necessary to address the taxonomic uncertainties and increase our knowledge of the diversity of Ampycidae.

## Supplementary Material

XML Treatment for Phalangodus

XML Treatment for Phalangodus andresi

XML Treatment for Ventrifurca

XML Treatment for Ventrifurca phallaina

XML Treatment for Ampycella

XML Treatment for Ampycella fortunata

## ﻿Additional information

### Author ORCIDs

Osvaldo Villarreal https://orcid.org/0000-0001-5355-3723

Daniela Ahumada-C. https://orcid.org/0000-0002-4182-5143

Leonardo Delgado-Santa https://orcid.org/0000-0002-9714-6924

### Data availability

All of the data that support the findings of this study are available in the main text or Supplementary Information.

## Supplementary materials

Supplementary material 1 List of Laniatores species for Colombia Data type xlsx Explanation note List of Laniatores species for Colombia, with their registration source, distribution associated with WWF biogeographic ecoregions and their presence in protected areas, National System of Protected Areas. https://binary.pensoft.net/file/893721This dataset is made available under the Open Database License (http://opendatacommons.org/licenses/odbl/1.0/). The Open Database License (ODbL) is a license agreement intended to allow users to freely share, modify, and use this Dataset while maintaining this same freedom for others, provided that the original source and author(s) are credited.Osvaldo Villarreal, Daniela Ahumada-C., Leonardo Delgado-Santa
